# Research Progress of Exosomes in Bone Diseases: Mechanism, Diagnosis and Therapy

**DOI:** 10.3389/fbioe.2022.866627

**Published:** 2022-04-12

**Authors:** Fanying Meng, Xu Xue, Zhifeng Yin, Fei Gao, Xiuhui Wang, Zhen Geng

**Affiliations:** ^1^ Institute of Translational Medicine, Shanghai University, Shanghai, China; ^2^ Department of Orthopedics, Shanghai Zhongye Hospital, Shanghai, China

**Keywords:** exosomes, bone diseases, micro-RNA, biomaterials, bone regeneration

## Abstract

With the global escalation of the aging process, the number of patients with bone diseases is increasing year by year. Currently, there are limited effective treatments for bone diseases. Exosome, as a vital medium in cell-cell communication, can mediate tissue metabolism through the paracrine transmission of various cargos (proteins, nucleic acids, lipids, etc.) carried by itself. Recently, an increasing number of researchers have proven that exosomes play essential roles in the formation, metabolism, and pathological changes of bone and cartilage. Because exosomes have the advantages of small size, rich sources, and low immunogenicity, they can be used not only as substitutes for the traditional treatment of bone diseases, but also as biomarkers for the diagnosis of bone diseases. This paper reviews the research progress of several kinds of cells derived-exosomes in bone diseases and provides a theoretical basis for further research and clinical application of exosomes in bone diseases in the future.

## Graphical Abstract

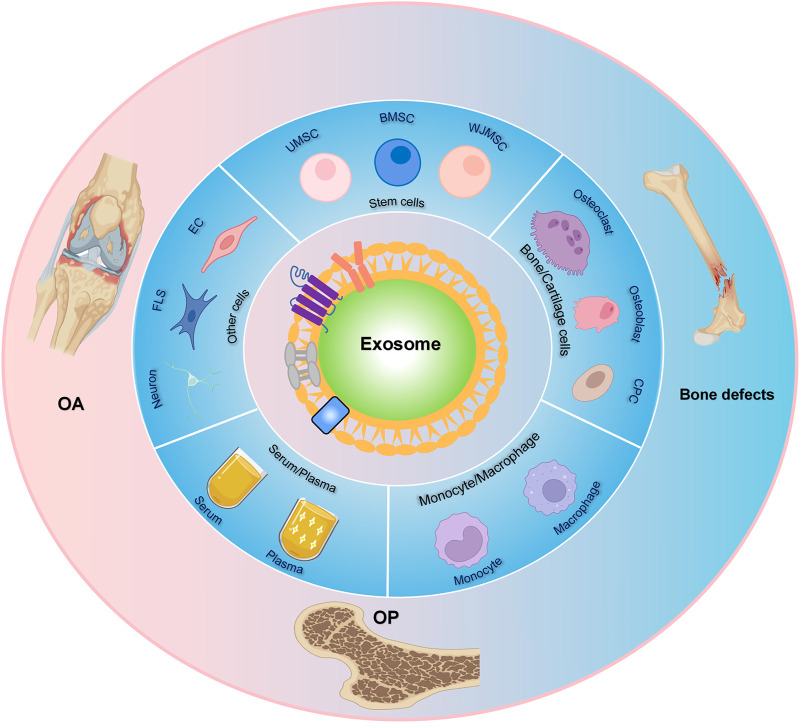

## Introduction

With the intensification of global aging, the number of patients with bone-related diseases (including osteoarthritis (OA), osteoporosis (OP), bone defect) has increased sharply (Zhao H. et al., 2018; Mei et al., 2020; Ren et al., 2021). These diseases not only bring physical and psychological pain to patients, but also result in enormous financial burdens to society (Hernlund et al., 2013; Hu et al., 2021a; Xue et al., 2021b). For OA, it was estimated that nearly 250 million OA patients worldwide, and the incidence was anticipated to increase further (Hunter and Bierma-Zeinstra, 2019). A statistical report pointed out that in some developed countries, the cost of treating OA could reach 1–2.5% of gross domestic product (Hunter et al., 2014). At present, the main ways to treat OA include drug treatment and surgical intervention (Zhou et al., 2020; Jin et al., 2021). However, painkillers and anti-inflammatory drugs commonly used in drug treatment could only relieve pain, not completely cure it. In addition, although advanced OA could be surgically intervened, it might be accompanied by many complications. In short, there is still no effective treatment to reverse the progression of OA (Glyn-Jones et al., 2015; Hu et al., 2019; Fang et al., 2021; Yajun et al., 2021). For OP, estimates showed that more than 200 million people worldwide suffered from OP (Ensrud and Crandall, 2017). Statistically, OP-related fractures cost approximately $17.9 and 4 pounds billion per year in the United States and United Kingdom, respectively (Clynes et al., 2020). Additionally, among hip fractures caused by OP, 21–30% of patients died within 1 year (Force et al., 2018). Unfortunately, OP usually takes a long period and even requires lifelong treatment with many side effects (Compston et al., 2019; Chen J. et al., 2021). For bone defects, although autogenous and allogeneic bone grafts have been widely used for repairing bone defects, there are still some limitations in these two treatments, such as inflammation, limited autogenous bone, and immune rejection (Lee and Goodman, 2015; Qin et al., 2016; Xue et al., 2021a; Chen W. et al., 2021). In recent years, stem cell treatment has been located at the forefront of the field of bone regenerative medicine (Hao et al., 2016; Debnath et al., 2018). However, its molecular mechanism is not precise, and there are still some limitations, such as high cost, limited donors, and so on. Therefore, alternative and novel treatment strategies are imperatively needed to meet the clinical requirements of bone diseases.

**FIGURE 1:**
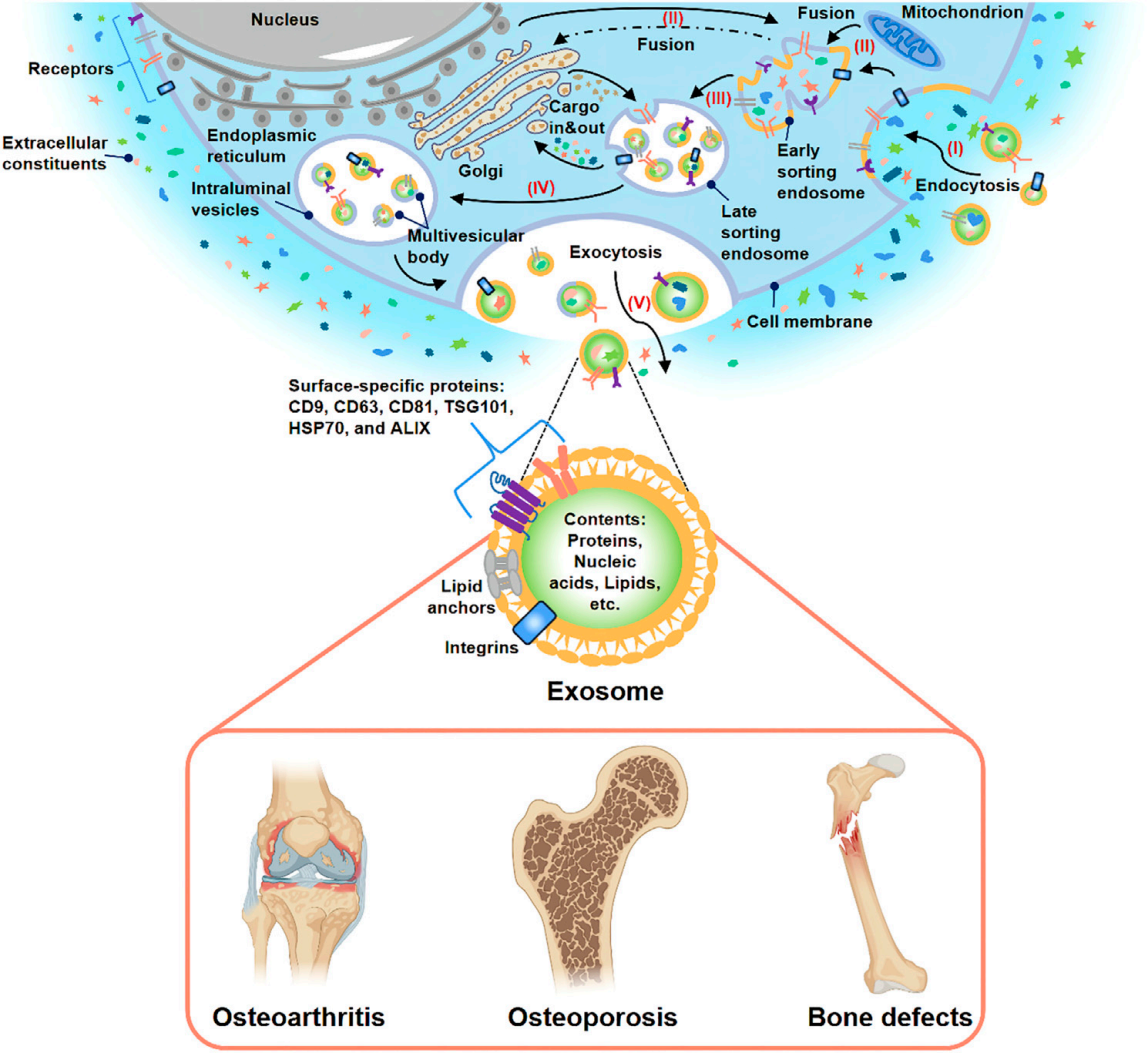
Biogenesis, secretion, and potential application of exosome. (I) Extracellular constituents and cell surface proteins entered cells through endocytosis. (II) The formation of the cell membrane budded in the luminal side. The fusion of the bud with the compositions of the endoplasmic reticulum, Golgi, and mitochondria gave rise to early sorting endosome formation. (III) Then, early sorting endosome led to late sorting endosome. (IV) The secondary invagination was modified with cargos, giving rise to the generation of various intraluminal vesicles and the formation of the multivesicular body. (V) At last, the exocytosis of the multivesicular body released intraluminal vesicles to the outside of cells in the form of exosomes. Many studies have demonstrated that exosome-based treatment was effective for OA, OP, and bone defects.

It is generally considered that bone is a complex organ containing various cells, such as osteoblasts, osteoclasts, macrophages, endothelial cells, stem cells, and other cells (Wang L. et al., 2021). These cells surrounding the bone microenvironment communicate with each other and participate in bone metabolism together. Extracellular vesicles (EVs) have attracted extensive attention as an important medium of cell-cell communication (van der Pol et al., 2012). EVs can be further divided into exosomes, ectosomes (microparticles/microvesicles), apoptotic bodies, oncosomes and some other EVs subtypes (Thery et al., 2018; Ni et al., 2020). Among these different subtypes of EVs, exosome is one of the most widely studied subtypes as evidenced by an exponentially increasing number of exosome-related researches in recent years (Lawson et al., 2016). Exosome has a phospholipid bilayer structure with a diameter of 30–150 nm and can be secreted by various cells through the endosomal pathway (Figure 1) (Thery et al., 2002; Klumperman and Raposo, 2014). The history of exosomes could be traced back to 1983. It was firstly found by Harding et al. (1983), Pan and Johnstone (1983) in rat and sheep animal models, respectively. They found that a nano-vesicle containing transferrin was released during the maturation of reticulocytes into erythrocytes. According to its endosomal origin, this nano-vesicle was officially named “exosome” by Johnstone in 1987 (Johnstone et al., 1987). Over a long period of time, exosomes were considered to be cellular waste used by cells to transport metabolic products and little attention was paid to them. In recent years, accumulating evidence indicated that exosome was a natural endogenous nano-carrier that played an essential role in cell-cell communication (Mathieu et al., 2019; Cheng et al., 2021). This was mainly due to the fact that exosomes could involve in cell signaling and intercellular communication by carrying various bioactive substances (including proteins, nucleic acids, lipids, etc.) to the target cells (Yang D. et al., 2020; Norouzi-Barough et al., 2021). Moreover, they could exist stably in body fluids (such as blood, urine and breast milk, etc.) and conditioned medium for cell culture (Han et al., 2016; Cobelli et al., 2017). Because of the above characteristics, Masaoutis et al. proposed that exosomes could act as an important signaling mediator in bone remodeling (Li Q. et al., 2018; Masaoutis and Theocharis, 2019). Nowadays, a large number of growth studies have demonstrated that exosomes could be used in cell-free therapy to effectively improve bone disease and solve the problems (immune rejection, insufficient bone mass, and poor stability) caused by traditional bone implants and stem cell therapy (Bjorge et al., 2017; Huang J. et al., 2021).

At present, the research field of EVs is not mature and still faces many challenges. Due to the problems of overlapping size, similar composition and lack of standardized surface markers, the existing EVs isolation methods (ultracentrifugation method, kit-based method, etc.) and purification methods could not prepare purified EVs specific subtypes. Therefore, the current EVs preparations obtained are highly heterogeneous. In order to further accelerate the regulation and development of this field, the International Society for Extracellular Vesicles (ISEV) issued the Minimal Information for Studies of EVs in 2018 (MISEV 2018) (Thery et al., 2018). MISEV2018 recommended naming different subtypes of EVs according to physical characteristics, biochemical composition, or cell of origin unless the pathway can be demonstrated by live imaging techniques. For the purposes of this review, “exosomes” are described as EVs with a diameter of 30–200 nm whose surface markers meet the basic requirements of MISEV2018 and regardless of their purity and origin. Based on the current knowledge, this article reviews the investigation status of exosomes in the domain of bone diseases and aims to bring novel approaches for bone diseases treatment in the clinic (Figure 1).

## Exosomes and Bone Diseases

### Stem Cell-Derived Exosomes

Stem cells are cells with a very high differentiation potential, which have been confirmed to play critical roles in the growth of bone (Jiang et al., 2020). Currently, stem cell therapy has been used in the clinic as an alternative strategy for allogeneic and allogeneic bone transplantation (Perez et al., 2018). Recent researches pointed out that the function of stem cells in promoting bone repair is mainly attributed to their paracrine secretion (Tao et al., 2018). Exosome had been proved to be an essential medium involved in paracrine. In this way, exosome, which could bypass immune rejection caused by stem cell therapy and bone deficiency in traditional surgical techniques, was regarded as a good candidate for cell-free therapy (Furuta et al., 2016; Hu et al., 2021b). In the last decades, stem cell-derived exosomes were demonstrated better therapeutic potential in OA, OP, and bone defects (Liu S. et al., 2020). The following paragraphs and Supplementary Table S1 introduce the effects of several stem cells-derived exosomes in bone diseases.

#### Bone Marrow Mesenchymal Stem Cell-Derived Exosomes

**FIGURE 2:**
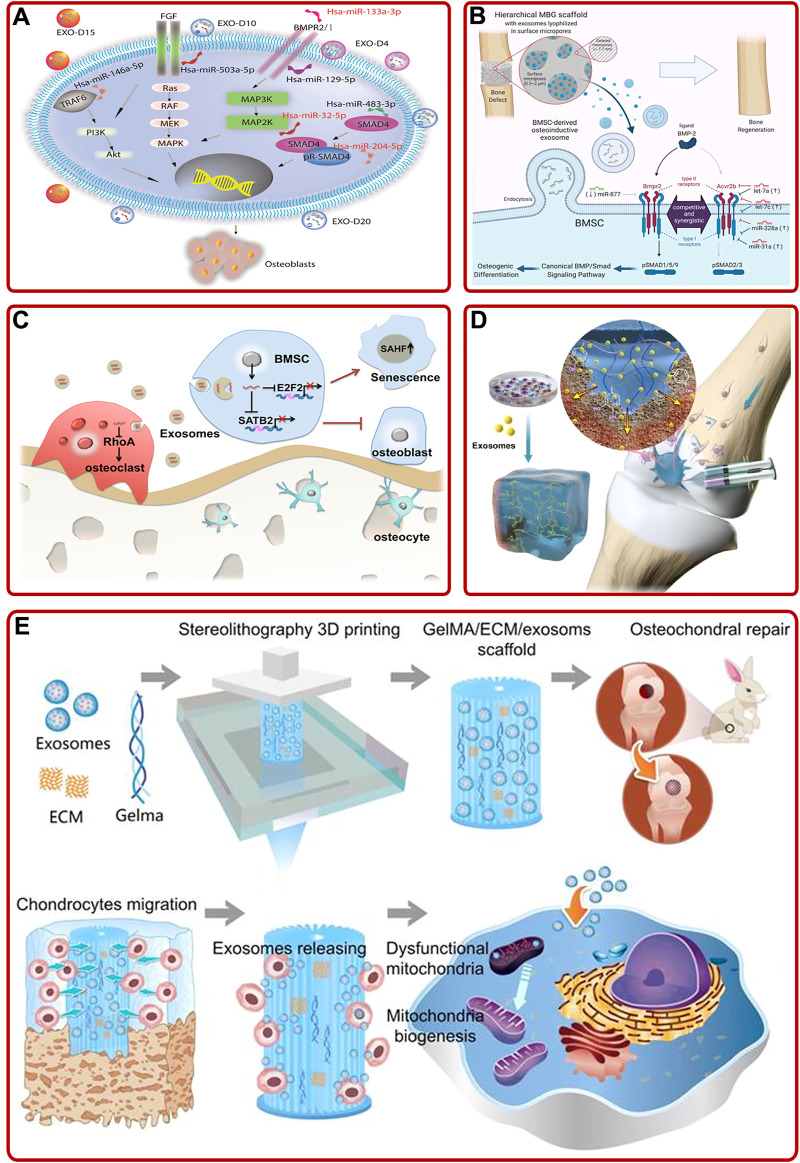
**(A)** BMSC-derived exosomes induced osteogenic differentiation of hMSCs through mediating the PI3K/Akt and MAPK signaling pathways by upregulating/downregulating osteogenic and anti-osteogenic miRNAs (Zhai et al., 2020). **(B)** Schematic of the mechanism BMSC-derived osteoinductive exosomes functionalized hierarchical MBG scaffold promoted bone regeneration (Liu A. et al., 2021). **(C)** Aged BMSC- derived exosomal miR-31a-5p inhibited BMSCs functions in cellular aging and osteogenesis *via* the E2F2 and SATB2 signaling pathways and promoted osteoclastic differentiation via the RhoA signaling pathway (Xu et al., 2018). **(D)** Schematic described that superficial cartilage could be regenerated by injecting BMSC-derived exosomes containing hydrogels (Zhang F.-X. et al., 2021). **(E)** 3D printed ECM/Gelma/BMSC-derived exosomes scaffold enhanced chondrocyte mitochondrial biogenesis and promoted osteochondral regeneration in rabbit models (Chen et al., 2019).

Bone marrow mesenchymal stem cells (BMSCs) mainly differentiate into osteoblasts and chondrocytes, so they have great effects on bone/cartilage formation (Fan et al., 2017; Gao et al., 2021). During this process of differentiation, exosomes activated and participated in different intracellular pathways to facilitate osteogenesis/chondrogenesis by transporting their cargos (Metavarayuth et al., 2019; Wong et al., 2020). For example, Zhang L. et al. (2020) elucidated that BMSC-derived exosomes improved fracture healing by promoting osteogenesis and angiogenesis via the BMP-2/Smad1/RUNX2 signaling pathway. Furthermore, the impacts of BMSC-derived exosomes on angiogenesis could be further enhanced by low-dose dimethyloxalylglycine (Liang et al., 2019). In addition, Zhao P. et al. (2018) indicated that BMSC-derived exosomes could proliferate osteoblasts and relieve OP through the MAPK signaling pathway. Among various cargos carried by exosomes, small RNA (sRNA), especially micro-RNA (miRNA), has been proved to be the primary medium involved in cell-cell communication (Pegtel et al., 2010). For instance, Zhai et al. (2020) concluded that human BMSC (hBMSC)-derived exosomes could facilitate bone formation by upregulating osteogenic miRNAs (Hsa-miR-146a-5p, Hsa-miR-503-5p, Hsa-miR-483-3p, and Hsa-miR-129-5p) or downregulating anti-osteogenic miRNAs (Hsa-miR-32-5p, Hsa-miR-133a-3p, and Hsa-miR-204-5p) to activate PI3K/Akt and MAPK signaling pathways (Figure 2A). Moreover, Fan et al. (2020) found that downregulation of miR-29a could inhibit the expression of natural bone morphogenetic protein (BMP) antagonist noggin, activate BMP/Smad signaling pathway, and enhance the osteogenic characteristics of BMSCs-derived exosomes. Additionally, Liu A. et al. (2021) optimized BMSC-derived exosomes immobilized in hierarchical mesoporous bioactive glass (MBG) scaffold via lyophilization. Then they found that BMSC-derived exosomal let-7a-5p, let-7c-5p, miR-328a-5p, and miR-31a-5p could activate the phosphorylation of Smad1/5/9 and activate BMP/Smad signaling pathway by targeting Acvr2b to mediate osteogenic differentiation and repair bone defects (Figure 2B). Besides, other BMSC-derived exosomal miRNAs, such as miR-122-5p (Liao et al., 2019), miR-15b (Li Y. et al., 2020), miR-206 (Huang et al., 2021d), miR-19b (Huang et al., 2021c), miR-146a (Liu L. et al., 2021), miR-877 (Liang et al., 2021), miR-218 (Hassan et al., 2012), and miR-128-3p (Xu et al., 2020) also played significant roles in stimulating osteogenesis and angiogenesis. Briefly, BMSCs-derived exosomal miRNAs could participate in various pathways to promote bone formation, suggesting BMSC may be a key cell in maintaining bone metabolism.

It is worth noting that not all BMSC-derived exosomes play an active role in promoting osteogenesis. A rat model of OP with alveolar bone degeneration study showed that BMSC-derived exosomes accelerated osteoclasts formation (Xu and Wang, 2017). In addition, serval studies have pointed out the effect of age on exosomes. Xu et al. (2018) demonstrated that overexpression of aged BMSC-derived exosomal miR-31a-5p could not only inhibit osteogenesis *via* the E2F2 and SATB2 signaling pathways, respectively, but also promote osteoclastic differentiation through the RhoA pathway, resulting in bone loss (Figure 2C). Meanwhile, Jia et al. (2020) provided convincing evidence that BMSC-derived exosomes from young rats could upregulate the expression of osteogenesis-related genes, promote BMSCs proliferation, and accelerate bone establishment in aged rats. The above studies suggested that special attentions should be paid to the effects of pathology, age and other internal or external factors when using exosomes to treat bone diseases in the future.

BMSC-derived exosomes also played crucial roles in promoting cartilage repair and relieving OA. On the one hand, BMSC derived-exosomes promoted cartilage repair by directly acting on chondrocytes or indirectly participating in cartilage-related signaling pathways. For example, Hingert et al. (2020) proposed that BMSC-derived exosomes could improve the activity of chondrocytes and accelerate chondrocytes proliferation. Further mechanism studies pointed out that BMSC-derived exosomal miR-136-5p facilitated chondrocyte migration and proliferation through targeting ELF3 and downregulating ELF3 expression in a traumatic OA mouse model (Chen et al., 2020e). In addition, BMSC-derived exosomal circular RNA (circRNA_0001236) could also facilitate cartilage repair through miR-3677-3p/Sox9 signaling axis (Mao et al., 2021). Interestingly, Liu et al. obtained an exosome *via* pretreating BMSC-derived exosome by a new small molecular compound Kartogenin. Then, they found that the obtained exosome could promote chondrogenesis through upregulating COL2A1, Prg4, and SOX-9 (Liu C. et al., 2020). On the other hand, OA could lead to changes in the extracellular matrix of cartilage and aggravate the injury due to inflammation. BMSC-derived exosomes maintained the stability of cartilage extracellular matrix and alleviate OA by inhibiting inflammatory factors or inflammation-related signaling pathways (Jin et al., 2020). For instance, He et al. (2020b) confirmed that BMSC-derived exosomes could promote cartilage formation by weakening the inhibitory effects of pro-inflammatory cytokines on chondrocyte proliferation and migration. Moreover, Vonk et al. (2018) found that BMSC-derived exosomes could affect the cartilage homeostasis, stimulate the production of polysaccharides and collagen, and inhibit the upregulation of pro-inflammatory interleukin. Additionally, BMSC-derived exosomes reduced cartilage injury via converting the M1 phenotype of macrophages to the M2 phenotype, decreasing the release of inflammatory factors (Zhang J. et al., 2020). Further research proved that mouse BMSC-derived exosomes overexpressing miRNA-210 could protect chondrocytes from injury by inhibiting the inflammation-related NF-κB signaling pathway (He et al., 2020a). Meanwhile, the treatment of rat OA models proved that low-intensity pulsed ultrasound could enhance this process (Liao et al., 2021).

Exosomes are often combined with technologies or biomaterials to achieve ideal functions, such as specific bone targeting, better osteogenic properties, and bone healing ability. For instance, Luo et al. (2019) fabricated an engineered exosome through coupling exosomes with BMSC-specific aptamers *via* high-affinity recognition. The engineered exosomes had a specific bone targeting ability and could significantly induce bone regeneration. In addition, Huang et al. (2020) prepared a functional exosome (BMP2 overexpression) via gene-editing technology. The exosome not only promoted the expression of BMP2, RUNX2, Osterix, and BMP9 *in vitro*, but also accelerated the healing of skull defects *in vivo*. In addition, other data indicated that exosomes and materials had a synergistic role in tissue healing. For example, Zhang F.-X. et al. (2021) found that BMSC-derived exosomes and adhesive hydrogel had a synergistic role in promoting BMSCs migration, proliferation, and differentiation, and repairing the osteochondral defects (Figure 2D). As well, Chen et al. (2019) demonstrated that chondrocyte extracellular matrix (ECM)/methacrylic acid gelatin (Gelma)/BMSC-derived exosomes scaffold fabricated by 3D printing technology could repair the mitochondrial dysfunction of chondrocytes, promote chondrocytes migration, and facilitate cartilage regeneration in rabbit models (Figure 2E). In addition, other studies suggested that BMSC-derived exosomes modified biomaterials, such as TiO2 nanotubes and hydrogel, exhibited superior properties on BMSCs proliferation and differentiation, inflammation regulation, and bone healing (Huang C.-C. et al., 2021; Zhang B. et al., 2021; Zhao et al., 2021). In brief, the addition of exosomes or engineered exosomes could prepare better biomaterials for the treatment of bone diseases, which pointed out a novel research direction.

#### Umbilical Cord Mesenchymal Stem Cell and Wharton’s Jelly of Umbilical Cord Mesenchymal Stem Cell-Derived Exosomes

**FIGURE 3:**
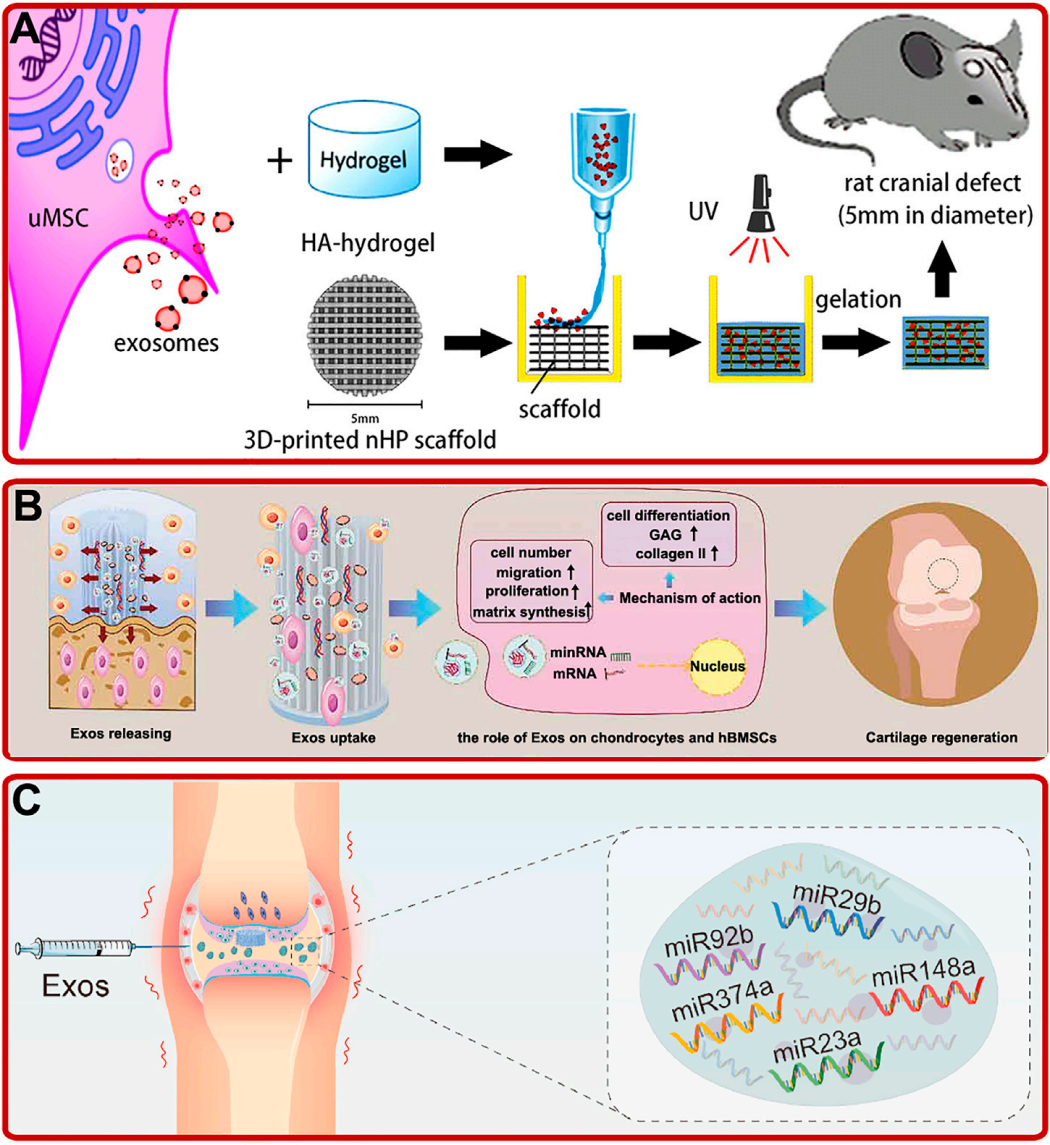
**(A)** UMSC-derived exosomes combined with the HA-hydrogel and the 3D-printed nHP scaffold for cranial defect repair (Zhang Y. et al., 2021). **(B)** Exosomes (exos) released from hydrogel improved the migration, proliferation, and chondrogenic differentiation of hBMSCs and enhanced the formation of glycosaminoglycan (GAG), ECM, and collagen II, and thus promoted cartilage regeneration (Hu H. et al., 2020). **(C)** Schematic of WJMSC-derived exosomal miRNAs facilitated osteochondral regeneration (Jiang et al., 2021).

Compared with other stem cells-derived exosomes, human umbilical cord mesenchymal stem cell (hUCMSC) and Wharton’s jelly of umbilical cord mesenchymal stem cell (WJMSC)-derived exosomes have the characteristics of clean source, strong proliferation and high immunity. Several studies showed that hUCMSC-derived exosomes could promote OP and bone fracture healing by boosting BMSCs proliferation, migration, osteogenic differentiation and angiogenesis *via* Wnt and miR-1263/Mob1/Hippo signaling pathways (Zhang Y. et al., 2019; Zhou et al., 2019; Liu W. et al., 2020; Yang B.-c. et al., 2020). Based on these characteristics, by injecting hUCMSC-derived exosomes intravenously into a mouse OP model, Hu Y. et al. (2020) revealed that hUCMSC-derived exosomes could prevent the decrease of bone mass and maintain bone strength by promoting bone formation, reducing bone resorption and fat accumulation. In addition, hUCMSC-derived exosomes-modified hydrogels also showed excellent osteogenic and chondrogenic effects. For instance, by fabricating a hUMSC-derived exosomes modified Gel/hyaluronic acid (nHP) scaffold, Zhang et al. found that the addition of hUMSC-derived exosomes promoted rat cranial defects healing through activating the miR-21/NOTCH1/DLL4 signaling pathway (Figure 3A) (Zhang Y. et al., 2021). In addition, studies by Wang L. et al. (2020) revealed that hUCMSC-derived exosomes could enhance the reparative effect of coralline hydroxyapatite/silk fibroin/glycol chitosan/difunctionalized polyethylene glycol self-healing hydrogel in SD rats with induced femoral condyle defect. Moreover, Hu H. et al. (2020) prepared a hUCMSC-derived exosomes modified Gelma/nanoclay hydrogel. The modified hydrogel could effectively boost the chondrocytes migration, proliferation, and differentiation and promote cartilage regeneration (Figure 3B).

Wharton’s jelly of umbilical cord mesenchymal stem cell (WJMSC), which has a strong ability of proliferation and differentiation, is a kind of MSCs extracted from the umbilical cord (Li et al., 2015). Recently, studies indicated that WJMSC-derived exosomes could also play important roles in bone reconstruction. For example, Jiang et al. (2021) confirmed that human WJMSC-derived exosomes could promote the proliferation of BMSCs and chondrocytes, facilitate the polarization of macrophages to M2 phenotype, reduce the inflammatory response, and regenerate bone and cartilage (Figure 3C). In addition, Kuang et al. (2019) found that human WJMSC-derived exosomes could inhibit osteocyte apoptosis through miR-21/PTEN/AKT signaling pathway *via* TUNEL and high-throughput RNA sequencing methods.

#### Other Stem Cells-Derived Exosomes

In addition to the widely studied stem cell exosomes mentioned above, other stem cells (including adipose-derived mesenchymal stem cell (AMSC), induced pluripotent stem cell (iPSC), etc.)-derived exosomes also have potential therapeutic applications in the field of bone diseases.

**FIGURE 4:**
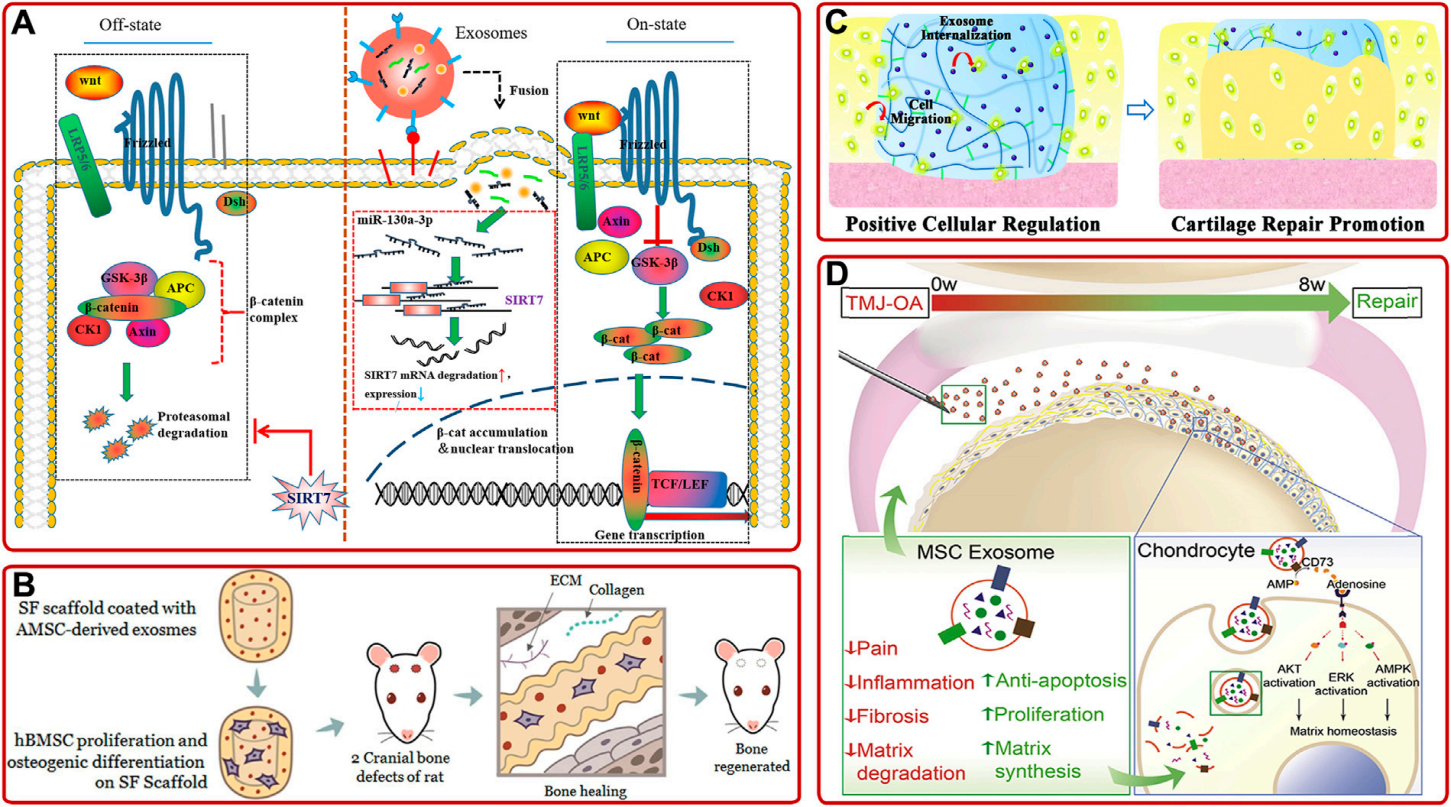
**(A)** Schematic diagram of the mechanism that AMSC-derived exosomal miR-130a-3p enhanced osteogenic differentiation of AMSCs via the SIRT7/Wnt/β-catenin signaling axis (Yang S. et al., 2020). **(B)** AMSC-derived exosomes-coated silk fibroin 3D-scaffold implants facilitated bone healing in the calvarial bone defects of SD rats (Kyung Kim et al., 2021). **(C)** IPSC-derived exosomes internalized hydrogel glue positively facilitated cells to promote cartilage repair (Liu et al., 2017). **(D)** Schematic of the proposed mechanisms of MSC-derived exosome in repairing and regenerating TMJ-OA (Zhang S. et al., 2019).

*AMSC-derived exosomes.* Previous studies showed that AMSC-derived exosomes could inhibit the activation of NLRP3 inflammasome in osteoclasts and reduce the apoptosis of osteoblasts to relieve OP (Ren et al., 2019; Wang S. et al., 2021; Zhang L. et al., 2021). Furthermore, Yang S. et al. (2020) demonstrated that miR-130a-3p played a vital role in determining the osteogenic differentiation of AMSCs-derived exosomes. They further proved that the overexpression of miR-130a-3p could downregulate the expression of SIRT7 and upregulate the expression of Wnt signaling pathway-associated protein (Figure 4A). Moreover, recent studies confirmed that AMSC-derived exosomes pretreated with TNF-α or osteogenic medium have better osteogenic ability than non-pretreated ones (Lu et al., 2017; Zhu et al., 2021). As well, AMSC-derived exosomes combined with biomaterials have been proved to promote osteogenic differentiation and bone regeneration cooperatively (Li W. et al., 2018; Li S. et al., 2021). For example, Kyung Kim et al. (2021) demonstrated AMSCs-derived exosomes modified silk fibroin 3D-scaffold showed better bone healing ability than non-modified scaffold in a calvarial bone defects SD rat model (Figure 4B).

*iPSC-derived exosomes.* IPSC can be induced from patients’ autologous stem cells and have no ethical problems, so they have attracted much attention. Meanwhile, emerging investigations have shown that iPSC-derived exosomes could provide a promising option in treating bone diseases. For example, Qi et al. (2016) found that exosomes secreted by MSCs derived from human induced pluripotent stem cells (hiPSC-MSC-Exos) could promote vascular and bone regeneration in ovariectomized (OVX) rat models. They also proved tricalcium phosphate modified with hiPSC-MSC-Exos could accelerate bone repair through activating PI3K/Akt signaling pathway (Zhang J. et al., 2016). In addition, other investigations showed that hiPSC-MSC-Exos had a stronger effect on facilitating chondrocytes migration and proliferation and ameliorating OA than synovial cells-derived exosomes (Zhu et al., 2017). This result opened up a way for IPSC-derived exosomes in cartilage repair, but the specific mechanism remained to be further studied. Based on this, Liu et al. (2017) developed a hydrogel glue with the incorporation of hiPSC-MSC-Exos by *in-situ* method (Figure 4C). Then, they found that the incorporation of hiPSC-MSC-Exos could significantly increase articular cartilage regeneration in a rabbit articular cartilage defect model.

Moreover, there are some other stem cells-derived exosomes, such as human embryonic stem cell-derived exosomes (Zhang S. et al., 2016; Zhang S. et al., 2018; Zhang S. et al., 2019), human gingival mesenchymal stem cell-derived exosomes (Diomede et al., 2018), human dental pulp stem cell-derived exosomes (Swanson et al., 2020; Xie et al., 2020), periodontal ligament stem cell-derived exosomes (Yu et al., 2021), human perivascular stem cell-derived exosomes (Xu et al., 2019), amniotic fluid stem cell-derived exosomes (Zavatti et al., 2020), and synovial mesenchymal stem cell-derived exosomes (Duan et al., 2021), has also been demonstrated to promote osteogenic differentiation, bone repair and cartilage repair. To be more specific, Zhang S. et al. (2019) found intravenous injection of hESC-MSCs-Exos could control pain and repair cartilage in temporomandibular joint OA (TMJ-OA). (Figure 4D). Especially, as the only known mammalian organ that can be regenerated annually, the deer antler is an ideal organ to overcome the insufficiency of stem cells. Lei et al. (2021) proved that the deer antler stem cell-derived exosomes could reduce inflammation, delay cell senescence, and promote bone and cartilage regeneration.

### Bone/Cartilage Cell-Derived Exosomes

**FIGURE 5:**
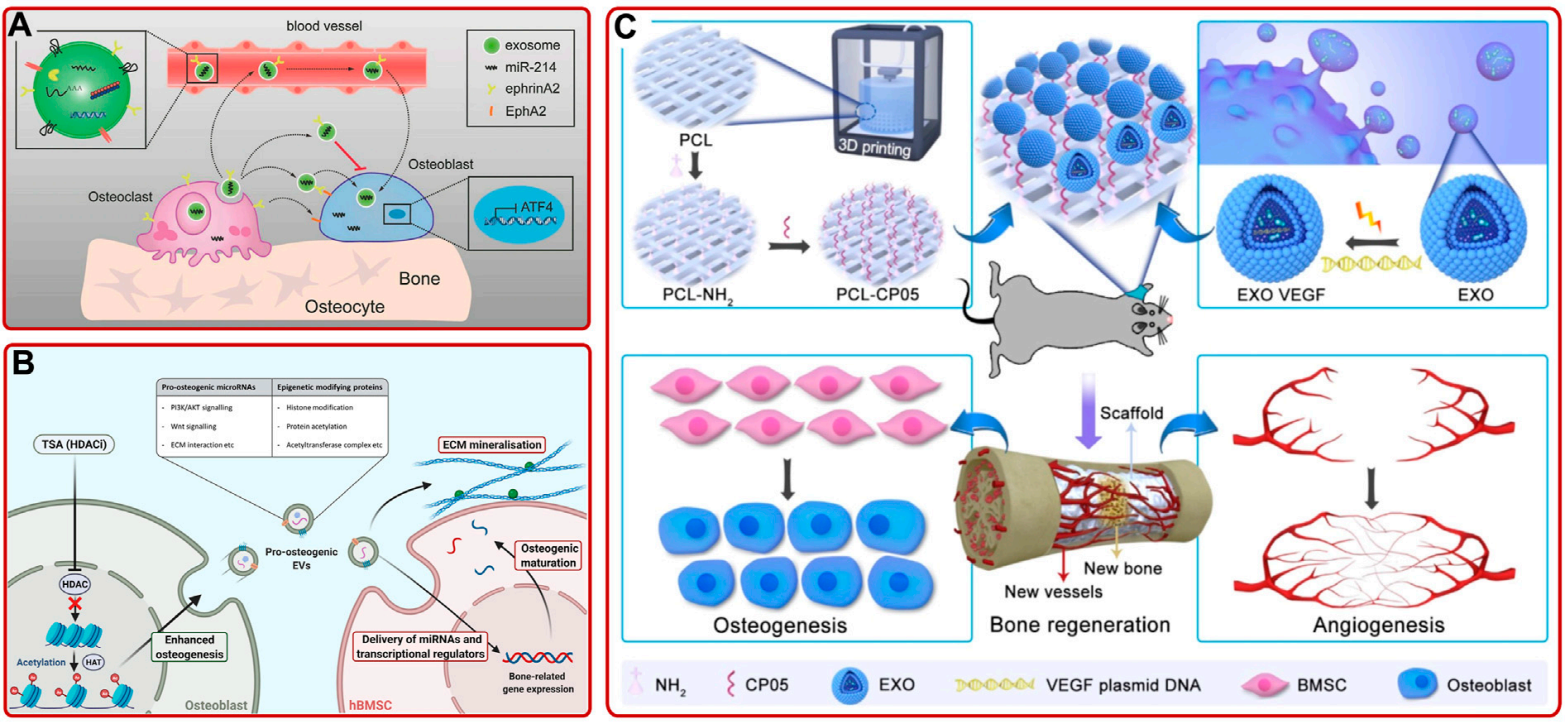
**(A)** MiR-214-containing exosomes from osteoclasts inhibited osteoblast function *via* ephrinA2/EphA2 recognition and can also be secreted into the blood as a biomarker for bone loss (Sun et al., 2016). **(B)** Schematic illustration of the mechanism that osteoblast-derived EVs pretreated with TSA enhanced the osteogenic differentiation of hBMSCs and osteoblast mineralization (Man et al., 2021). **(C)** 3D-printed porous PCL scaffold functionalized with VEGF@CPC-derived exosomes exhibited better osteogenic and angiogenic ability (Zha et al., 2021).

Osteoblasts and osteoclasts are the two most dominant cells that responsible for bone formation and resorption in the skeletal system, respectively (Yuan et al., 2016; Chen H. et al., 2020; Chen et al., 2020f). Osteoblast-osteoclast communication plays a key role in maintaining skeletal metabolism (Yuan et al., 2018). They coordinate with each other to maintain the homeostasis of the bone microenvironment (Deng et al., 2015; Chen et al., 2018a; Chen et al., 2018b; Li X. et al., 2020). Therefore, exploring the osteoblast-osteoclast communication will help understand the mechanism of bone homeostasis and provide a potential therapy for bone diseases treatment. Some studies suggested that exosomal miRNA took a crucial effect in osteoblast-osteoclast communication (Yin et al., 2017; Teotia et al., 2021). Sun et al. (2016) found that osteoclast-derived miR-214-containing exosomes could be secreted into the blood and inhibit the function of osteoblasts through interacting with ephrinA2 and EphA2 (Figure 5A). As well, Li et al. (2016) confirmed that osteoclast-targeted miR-214-3p inhibition augmented bone formation in aging OVX mice. On the contrary, osteoblast-derived exosomes could downregulate the heparanase gene and inhibit the differentiation of osteoclasts by increasing the expression of miR-503-3p during bone mineralization (Wang Q. et al., 2021). In addition, Ge et al. provided convincing evidence that osteoblast-derived exosomes contained osteogenesis-related proteins and could increase the number of bone trabeculae and bone volume in OVX rabbits (Ge et al., 2015; Ge et al., 2017; Sadat-Ali et al., 2021). Moreover, by transferring miRNA to activate the Wnt signaling pathway, osteoblast-derived exosomes could enhance the osteogenic differentiation of BMSCs (Cui et al., 2016). Additionally, osteoblast-derived EVs pretreated with histone deacetylase inhibitor trichostatin A (TSA) could significantly promote the osteogenic differentiation of hBMSCs and osteoblast mineralization (Figure 5B) (Man et al., 2021). These studies provided theoretical foundations for the clinical application of osteoblast-derived exosomes in the future. Surprisingly, Niedermair et al. (2020) showed that osteoblast-derived exosomes from patients with hip arthritis (CA), OP, and CA/OP could decrease BMSCs viability and alkaline phosphatase gene expression. This result suggested that the microenvironment of osteoblast-derived exosomal donor cells should be considered to avoid the opposite therapeutic effect.

Chondrogenic progenitor cell (CPC) is vital to maintain cartilage homeostasis and has received widespread attention in cartilage therapy (Kim et al., 2019). Currently, researchers have illustrated that CPC-derived exosomes play an essential part in MSC-chondrocyte communication and cartilage regeneration (Koelling et al., 2009; Yao and Wang, 2013). For example, Wang R. et al. (2020) discovered that CPC-derived exosomes not only enhanced chondrocytes proliferation and migration *in vitro*, but also promoted articular cartilage repair *in vivo*. In addition, several studies have shown that CPC-derived exosomes could also take effect in the therapy of bone defects. For instance, Zha et al. (2021) fabricated a VEGF@CPC-derived exosomes functionalized 3D-printed porous polycaprolactone (PCL) scaffold and verified that the functionalized scaffolds could induce osteogenic differentiation and promote angiogenesis and bone regeneration (Figure 5C). In contrast, current study showed that chondrocyte-derived exosomes could promote CPC proliferation, increase chondrogenesis markers’ expression, and inhibit angiogenesis (Chen Y. et al., 2018). The multiple effects of bone/cartilage cell-derived exosomes in bone diseases had been listed in Supplementary Table S2.

### Macrophage-Derived Exosomes

Macrophage-MSC communication plays a significant role in maintaining bone homeostasis and promoting bone establishment (Pieters et al., 2019; Chen K. et al., 2020; Kang et al., 2020; Wang D. et al., 2021). Previous studies have confirmed that macrophages communicate with MSC and affect its osteogenic differentiation through exosomes (Ekstrom et al., 2013). Moreover, recent studies demonstrated that exosomes derived from M0, M1, and M2 macrophages exerted distinct influences on the proliferation and differentiation of MSCs (Xia et al., 2020). For instance, researchers proved that M2 polarized macrophage-derived exosomes could inhibit the adipogenesis of BMSCs and promote its osteogenesis by mediating the miR-690/IRS-1/TAZ signaling axis (Li Z. et al., 2021). In another pathway, M2 polarized macrophage-derived exosomes could directly target salt-inducible kinase 2 and 3 genes to induce MSCs osteogenic differentiation (Xiong et al., 2020). In the clinic, it is universally acknowledged that diabetic patients often have a high incidence of fracture and prolonged healing time. A recent study illustrated that diabetic bone marrow macrophage-derived exosomes could inhibit the osteogenic differentiation of BMSCs and bone healing (Zhang D. et al., 2021). This inhibitory effect could be counteracted by inhibiting the expression of the miR-144-5p gene, which provided a new target for treating diabetic patients. Meanwhile, Bai et al. (2020) illuminated that macrophage-derived exosomes also have an influence on the growth of chondrocytes. Macrophage (pretreated with anti-inflammatory factor IL-4 or IL-13 *in vitro*) derived-exosomes, which containing Sox9 mRNA and protein, were beneficial to chondrocyte differentiation.

Based on the above functions of macrophage-derived exosomes, many studies combined them with biomaterials to improve osteogenic activity. For instance, Zhang T. et al. (2021) indicated that macrophage-derived exosomes modified titanium fostered osteoblast differentiation and mineralization, and osseointegration (Wei et al., 2019). Also, Liu et al. found that mineralized collagen pretreated with macrophage-derived exosomes could significantly facilitate MSC osteogenesis and bone regeneration (Liu A. et al., 2020). All these studies develop a novel way in the field of bone disease treatment. Supplementary Table S3 summarized some recent studies on macrophage-derived exosomes in bone diseases.

### Serum and Plasma-Derived Exosomes

**FIGURE 6:**
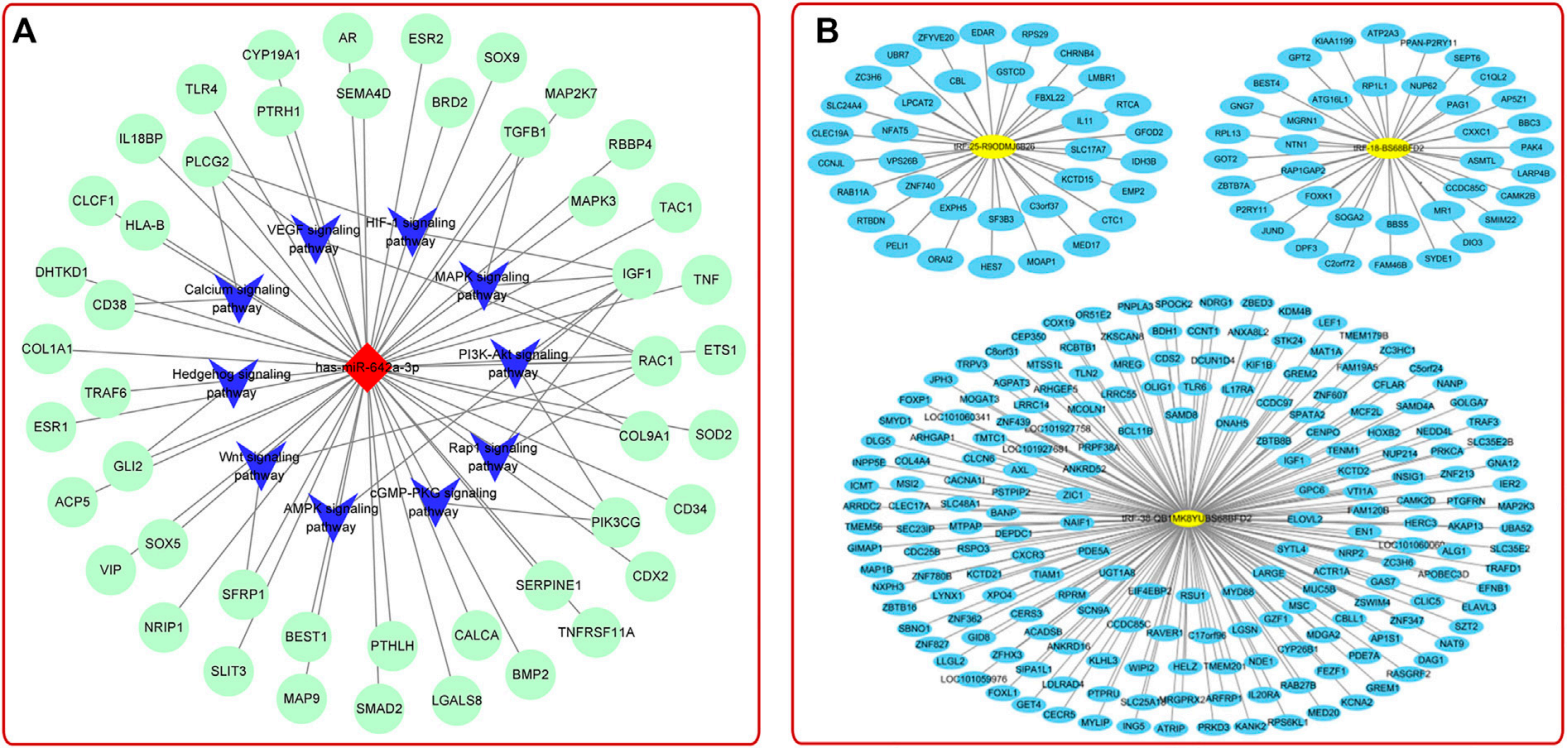
**(A)** MiRNA-mRNA-KEGG interaction network analysis showed the top 10 OP-related pathways (Kong et al., 2021). **(B)** TRF-25, tRF-38, and tRF-18 were potential targets to OP treatment (Zhang Y. et al., 2018).

There are a large number of exosomes in serum/plasma, and increasing studies pointed out that serum/plasma-derived exosomes have a great application prospect in early detection, diagnosis, and later treatment of bone diseases (Xie et al., 2018; Yang et al., 2021) (Supplementary Table S4). It is generally recognized that postmenopausal women often miss the best time for treating OP. In order to solve this problem, the researchers detected the plasma-derived exosomes of early postmenopausal women by small RNA sequencing method and concluded that exosomal miR-642a-3p might contribute to the prediction and diagnosis of early postmenopausal OP (Figure 6A) (Kong et al., 2021). Furthermore, Chen et al. found that plasma-derived exosomal TRF-25, TRF-38, and TRF-18 were closely related to OP (Figure 6B **)** (Zhang Y. et al., 2018). These studies use cutting-edge bioinformatics, and the results obtained by big data’s analysis were provided references for further exploration of the pathological mechanism of OP. In addition, other studies have shown that plasma-derived exosomal surface protein markers (PSMB9, AARS, PCBP2, and VSIR) (Chen M. et al., 2020), serum-derived exosomal hsa_circ_0006859 (Zhi et al., 2021), and long non-coding RNAs (LncRNA) (Teng et al., 2020) could also be used as new markers for the diagnosis of OP. Recently, studies found that the serum-derived exosomal miRNAs varied with age, external stimulation, and other factors. For example, serum-derived exosomes in young mice with high expression of miRNA-19b-3p could boost the BMSCs osteogenic differentiation in fatigue aged osteoporotic mice, suggesting that the osteogenic capacity of serum-derived exosomes was related to the age of the donor (Xun et al., 2021). In addition, Du et al. found that radiation could affect bone metabolism and regeneration by regulating the expression of plasma-derived exosomal miRNAs (Du et al., 2021). This study provided a novel method for the treatment of radiation-induced bone diseases.

### Other Exosomes

**FIGURE 7:**
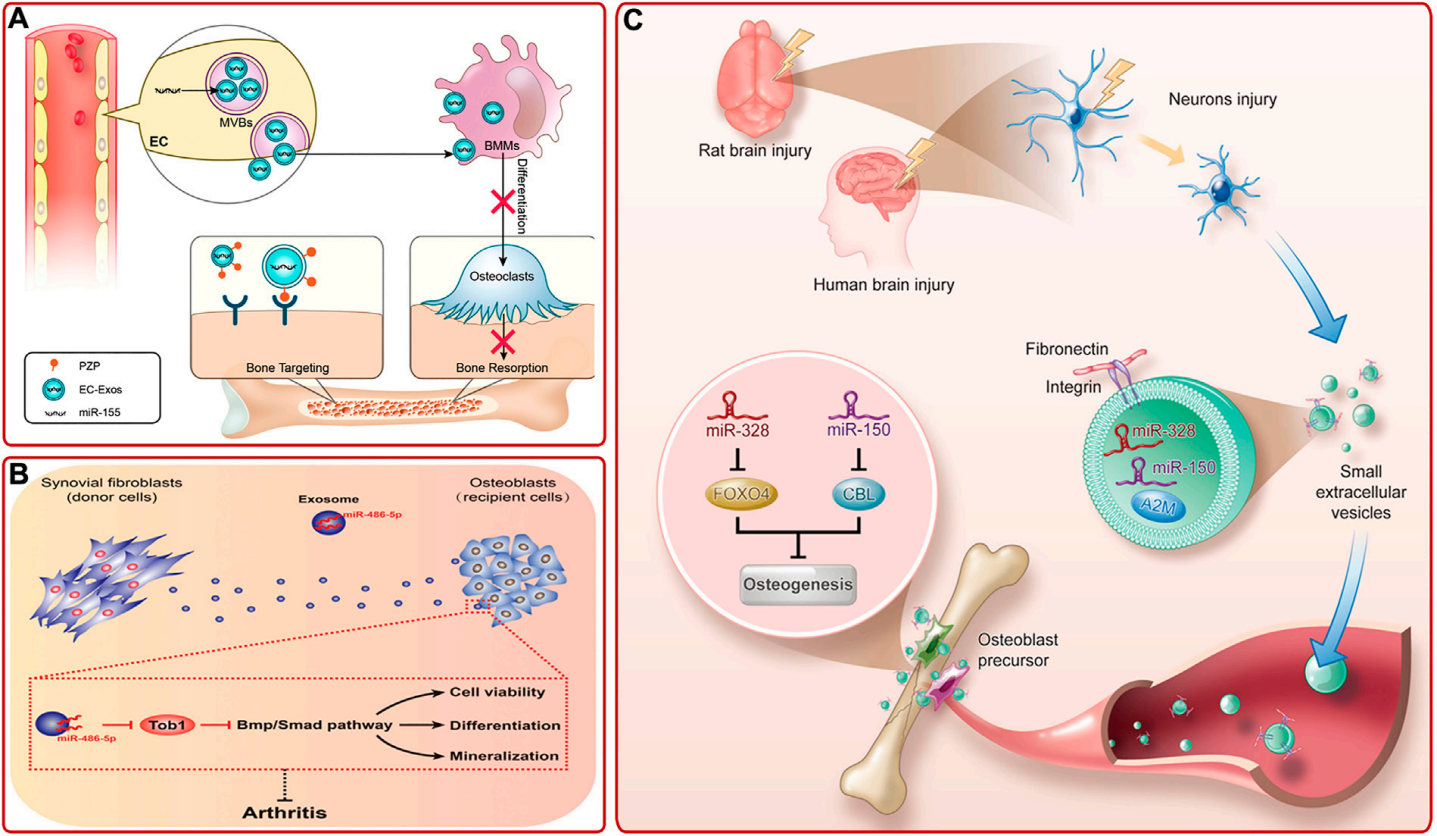
**(A)** Diagram of bone targeting and functional EC-exos (Song et al., 2019). **(B)** FLSs-exos carrying miR-486-5p inhibited Tob1 to activate the BMP/Smad signaling pathway, thus enhancing osteoblast proliferation, mineralization, and differentiation, then alleviating RA (Chen J. et al., 2020). **(C)** Schematic mechanism of bone healing accelerated by the damaged brain. Damaged neurons released exosomes, which were rich in miR-328a-3p and miR-150-5p. They could target FOXO4 and CBL and thus promoted osteogenesis (Xia et al., 2021).

Studies on other exosomes also provided multiple strategies for bone diseases treatment (Supplementary Table S5). For example, Sun et al. (2019) pointed out that sinus mucosa-derived cell-derived exosomes and periosteum-derived cell-derived exosomes could mediate paracrine effects to promote osteogenesis. The endothelial cell (EC) is a kind of highly active cells located in the inner layer of blood vessels, which can secrete a variety of substances and is essential for organogenesis (Seton-Rogers, 2014). Song et al. (2019) provided evidence that EC-derived exosomes (EC-exos) contained miR-155 and could reduce the activity of osteoclasts and promote bone targeting (Figure 7A). Recently, studies proved that endothelial progenitor cells (EPCs), which are precursors of ECs, could also indirectly promote bone formation *via* secreting exosomes. EPC-derived exosomes could facilitate bone formation by stimulating angiogenesis or by promoting the recruitment and differentiation of osteoclast precursors *via* LncRNA-MALAT1 (Cui et al., 2019; Jia et al., 2019). Fibroblast-like synoviocytes (FLSs) are the most important cells at the pannus-cartilage junction (Bustamante et al., 2017). Recent studies showed that FLSs-derived exosomes could also stimulate bone growth (Tan et al., 2020). For instance, Chen J. et al. (2020) found that FLSs-derived exosomes (FLSs-exos) could be swallowed by osteoblasts in the mouse model of collagen-induced rheumatoid arthritis (RA) and activate osteogenesis-related signaling pathways by upregulating miR-486-5p and targeting Tob1 to promote osteogenic differentiation (Figure 7B). Clinically, there was an interesting phenomenon that concomitant traumatic brain injury could accelerate bone formation. To explore the underlying mechanism, Xia et al. (2021) revealed that damaged neurons released exosomes (rich in miR-328a-3p and miR-150-5p) could facilitate osteogenesis by targeting the 3′UTR of FOXO4 and CBL (Figure 7C).

## Conclusion and Outlooks

Collectively, exosome is a natural nano-carrier containing various active cargos (miRNA, proteins, lipids, etc.) and plays a vital role in regulating bone metabolism. Based on their small size, wide source, and low immunogenicity, exosomes will have promising study and application prospects in the field of early diagnosis and therapy of bone diseases.

However, this field is still in the immature stage of basic research and preclinical exploration. The contents of exosomes are complex, which hinders the further exploration of their action mechanisms. It is worth mentioning that with the continuous development of proteomics and bioinformatics technology, several studies have been carried out more in-depth. For example, Chen M. et al. (2020) found four proteins closely related to OP (PSMB9, PCBP2, VSIR, and AARS) by quantitative proteomics and bioinformatics. This contributes to understanding the mechanism of bone disease. As the contents of exosomes are also related to different pathological conditions, external and internal factors, it is necessary to pay attention to the changes of conditions when studying the effects of different contents. In addition, a standardized separation and purification scheme is needed to further promote the clinical development of therapeutic exosomes. Consistent separation schemes and purification indexes are the premises of large-scale mass production for clinical application. The existing methods for the separation of exosomes include ultracentrifugation, commercial kit, size exclusion chromatography (SEC), tangential flow filtration (TFF), etc. Ultracentrifugation is the earliest and most widely used method. However, this method takes a long time and is difficult to meet the requirements of purity and yield. The commercial kit-based method takes less time than the ultracentrifugation method, but the specific principles and ingredients of most products have not been clarified. Recently, SEC and TFF based on size separation have been recognized by increasing researchers. Because it can obtain higher purity exosomes more easily. However, it is necessary to remove serum from the conditioned medium, which may change the activity of donor cells. In order to establish a perfect preparation system, Lim et al. proposed a set of relatively optimal manufacturing processes for researchers’ reference (Reiner et al., 2017). For instance, the system should have the characteristics of high capacity (unless the product is high potency), high yield, and reproducible purity. Meanwhile, the system should be a closed system with clear reaction principles and disposable components, and accord with serum-free culture. In addition, the manufacturing process should follow the Good Manufacturing Practice, which requires scientists to work together to establish a standard manufacturing process. Currently, another challenge for the clinical promotion of therapeutic exosomes is that they have not been strictly tested for clinical potency. In order to solve this problem, Lim et al. proposed a series of potentially quantifiable indicators to reach a consensus on the potency of different preparations (Witwer et al., 2019). Additionally, they proposed that different indicators should be evaluated by different independent laboratories and then analyzed and discussed together to ensure their credibility. In order to accelerate the application of exosomes, the cooperation of different teams is necessary. Furthermore, researchers can detect specific components that play vital roles in different diseases. Lim et al. proposed that under the guidance of major regulators, exosomes could be detected by a specific component to ensure that the potency of exosomes in the clinical treatment process is consistent (Gimona et al., 2021). In addition, the mode of exosomes’ administration (systemic or local) needs to be considered. Previously, most studies used systemic injections. Recent studies combined exosomes with biomaterials to locally administer drugs to animals. These results showed excellent effects of promoting bone and cartilage formation. However, the difference between systemic administration and local administration in the treatment of bone diseases needs to be further studied. In particular, due to the above limitations, few studies have compared the effects of different sources-derived exosomes in bone diseases treatment. Personalized treatment for different diseases is gaining importance. It is hoped that after standardizing therapeutic exosomes, there will be increasing studies to analyze the therapeutic effects of different exosomes. Briefly, it will be a trend to study exosomes in the future, which will help to broaden our cognitive field of exosomes and provide more strategies for the treatment of bone diseases.
