# Supplementary material for: Research Progress of Exosomes in Bone Diseases: Mechanism, Diagnosis and Therapy
Source: Front Bioeng Biotechnol. 2022 Apr 12;10:866627. doi: 10.3389/fbioe.2022.866627 (PMC9039039; doi:10.3389/fbioe.2022.866627)
Supplement: Supplementary file 1 [file DataSheet1.docx]

**Table S1.** Stem cells-derived exosomes

| **Sources** | **MiRs** | **Functions** | **Model system** | **Application** | **Ref** |
| --- | --- | --- | --- | --- | --- |
| BMSCs | - | Facilitate fracture healing | Mouse femur fracture models | Bone fracture | (Furuta et al., 2016) |
| NIH-3T3 cells | - | Promote osteogenesis and inhibit adipogenesis of BMSCs | Mouse age-related OP models | OP | (Hu et al., 2021) |
| BMSCs | - | Promote cartilage repair | Rabbit osteochondral defect models | OA | (Wong et al., 2020) |
| BMSCs | - | Promote osteogenesis and angiogenesis | Rat nonunion models | Bone fracture | (Zhang et al., 2020b) |
| BMSCs | - | Stimulate angiogenesis | Rat calvarial defect rat models | Bone defect | (Liang et al., 2019) |
| BMSCs | - | Promote osteoblast proliferation | HFOB 1.19 cells | OP | (Zhao et al., 2018) |
| BMSCs | Hsa-miR-146a-5p, Hsa-miR-503-5p, Hsa-miR-483-3p, Hsa-miR-32-5p,  Hsa-miR-133a-3p, Hsa-miR-204-5p | Induce the osteogenic differentiation by upregulating osteogenic miRNAs (Hsa-miR-146a-5p, Hsa-miR-503-5p, Hsa-miR-483-3p, and Hsa-miR-129-5p) or downregulating anti-osteogenic miRNAs (Hsa-miR-32-5p, Hsa-miR-133a-3p, and Hsa-miR-204-5p) | Rat radial bone defect models | Bone defect | (Zhai et al., 2020) |
| BMSCs | MiR-29a | Enhance osteogenesis via inhibition of miR-29a | Mouse calvarial defect models | Bone defect | (Fan et al., 2020) |
| BMSCs | Let-7a-5p,  let-7c-5p,  miR-328a-5p,  miR-31a-5p | Enhance the bone-forming capacity and induce bone regeneration | Rat calvarial defect rat models | Bone defect | (Liu et al., 2021a) |
| BMSCs | MiR-122-5p | Facilitate the proliferation and differentiation of osteoblasts | Rabbit osteonecrosis of the femoral head rabbit models | Osteonecrosis of the femoral head | (Liao et al., 2019) |
| BMSCs | MiR-15b | Promote osteogenic differentiation | Ovariectomized rat models | Bone defect | (Li et al., 2020) |
| BMSCs | MiR-206 | Facilitate osteoblast proliferation and differentiation | Mouse OA models | OA | (Huang et al., 2021c) |
| BMSCs | MiR-19b | Facilitate fracture healing | Mouse Femur fracture models | Bone fracture | (Huang et al., 2021b) |
| BMSCs | MiR-146a | Promote osteogenesis and angiogenesis | Rat distal femur defect models | Bone defect | (Liu et al., 2021b) |
| BMSCs | MiR-877 | Increase bone morphogenetic protein 2 expressions in hypoxic conditions and promote neoangiogenesis | Rabbit femoral condyle defect models | Bone defect | (Liang et al., 2021) |
| BMSCs | MiR-218 | Promote differentiation of osteoblasts and osteomimicry of metastatic cancer cells | MC3T3 osteoblasts, MDA-MB-231 metastatic breast cancer cells | Bone tumor | (Hassan et al., 2012) |
| BMSCs | MiR-128-3p | Inhibit bone fracture healing | Rat femoral fracture models | Bone fracture | (Xu et al., 2020) |
| **Sources** | **MiRs** | **Functions** | **Model system** | **Application** | **Ref** |
| BMSCs | - | Accelerate osteoclastogenesis in alveolar bone deterioration | Rat OP models | Alveolar bone deterioration | (Xu and Wang, 2017) |
| BMSCs | MiR-31a-5p | Promote osteoclastogenesis and bone resorption | Ovariectomized rat models | OP | (Xu et al., 2018) |
| BMSCs | - | Enhance the proliferation and osteogenic capacity of BMSCs | Rat tibial distraction osteogenesis models | Bone defect | (Jia et al., 2020) |
| BMSCs | - | Recruit BMSCs migration and inflation, promote BMSCs proliferation and differentiation | Rat osteochondral defect models | OA | (Zhang et al., 2021c) |
| BMSCs | - | Restore chondrocyte mitochondrial  dysfunction, enhance chondrocyte migration, and polarize the synovial macrophage response toward an M2 phenotype | Rabbit osteochondral defect models | OA | (Chen et al., 2019) |
| BMSCs | - | Improve cell viability and expedite chondrogenesis in disc cells | 3D human degenerative disc cell cultures | Intervertebr-al disc | (Hingert et al., 2020) |
| BMSCs | MiR-136-5p | Promote the migration of chondrocyte, increase collagen II, aggrecan, and SOX9 expression, reduce MMP-13 expression | Post-traumatic OA mouse models | OA | (Chen et al., 2020c) |
| BMSCs | CircRNA_0001236 | Promote cartilage-specific gene and protein expression, alleviate cartilage degradation, suppress OA progression, enhance cartilage repair | Mouse OA models | OA | (Mao et al., 2021) |
| BMSCs | - | Enhance the chondrogenic phenotype of chondrocytes, promote cartilage regeneration | Rat OA models | OA | (Liu et al., 2020b) |
| BMSCs | MiR-26a-5p | Retard damage of synovial fibroblasts, alleviate OA damage | Rat OA models | OA | (Jin et al., 2020) |
| BMSCs | - | Promote cartilage repair and extracellular matrix synthesis, alleviate knee pain in the OA rats | Rat OA models | OA | (He et al., 2020b) |
| BMSCs | - | Inhibit the adverse effects of inflammatory mediators on cartilage homeostasis, promote cartilage regeneration | OA patients’ chondrocytes | OA | (Vonk et al., 2018) |
| BMSCs | - | Alleviate cartilage damage, reduce osteophyte formation and synovial macrophage infiltration, inhibit M1 macrophage production and promote M2 macrophage generation. | Rat OA models | OA | (Zhang et al., 2020a) |
| BMSCs | MiR-210 | Improve the proliferation of chondrocytes, inhibit LPS-induced cell apoptosis | Lipopolysaccharide induced chondrocytes injury | OA | (He et al., 2020a) |
| BMSCs | - | Promote cartilage regeneration, increase chondrocyte proliferation and extracellular matrix synthesis, suppress inflammation, and inhibit the interleukin-1β-induced activation of the nuclear factor kappa B pathway | Rat OA models | OA | (Liao et al., 2021) |
|  |  |  |  |  |  |
| **Sources** | **MiRs** | **Functions** | **Model system** | **Application** | **Ref** |
| BMSCs | - | Enhance osteoblastic differentiation of BMSCs, promote bone regeneration | Ovariectomized mouse models | OP | (Luo et al., 2019) |
| BMSCs | - | Increase osteogenic differentiation and bone regenerative potential | Rat calvarial bone defect models | Bone defect | (Huang et al., 2020) |
| BMSCs | - | Promote bone repair and bone regeneration | Rat calvarial bone defect models | Bone defect | (Huang et al., 2021a) |
| BMSCs | - | Promote osteogenesis and angiogenesis | Rat posterolateral spinal fusion models | Bone defect | (Zhang et al., 2021a) |
| BMSCs | - | Enhance the migration and osteogenic differentiation of hBMSCs, show an immuno-regulatory role for macrophages | HBMSCs, macrophages | OA | (Zhao et al., 2021) |
| HUCMSCs | - | Enhance fracture healing through HIF‐1α‐mediated promotion of angiogenesis | Rat femoral fracture models | Bone fracture | (Zhang et al., 2019b) |
| HUCMSCs | - | Enhance fracture healing by increasing the protein expressions of β-catenin and Wnt3a in the Wnt signaling pathway | Rat femoral fracture models | Bone fracture | (Zhou et al., 2019) |
| HUCMSCs | MiR-216 | Facilitate bone fracture healing by promoting proliferation, migration, and tube formation in HUVECs, | Mouse femoral fracture models | Bone fracture | (Liu et al., 2020c) |
| HUCMSCs | MiR-1263 | Ameliorate disuse OP by inhibiting BMSCs apoptosis | Rat disuse OP models | OP | (Yang et al., 2020) |
| HUCMSCs | - | Prevent bone loss and maintain bone strength by enhancing bone formation, reducing marrow fat accumulation and decreasing bone resorption | Ovariectomized mouse models | OP | (Hu et al., 2020b) |
| HUCMSCs | MiR-21 | Accelerate bone repair by enhancing angiogenesis | Rat calvarial bone defect models | Bone defect | (Zhang et al., 2021f) |
| HUCMSCs | - | Promote bone repair by facilitating osteogenic differentiation of osteoblast progenitor cells and proliferation and migration of human umbilical vein endothelial cells | Rat femoral condyle defect models | Bone fracture | (Wang et al., 2020a) |
| HUCMSCs | MiR-23a-3p | Promote chondrogenesis by promoting the migration, proliferation and differentiation of chondrocytes and BMSCs | Rat cartilage defect models | Cartilage  defects | (Hu et al., 2020a) |
| WJMSCs | - | Promote osteochondral regeneration by enhancing the migration and proliferation of BMSCs and chondrocytes and promoting the polarization of macrophages toward the M2 phenotype | Rabbit and rat knee joint osteochondral defect models | OA | (Jiang et al., 2021) |
| WJMSCs | MiR-21 | Prevent glucocorticoid-induced osteonecrosis of the femoral head by inhibiting osteocyte apoptosis | Rat osteonecrosis of the femoral head models | Osteonecrosis of the femoral head | (Kuang et al., 2019) |
| **Sources** | **MiRs** | **Functions** | **Model system** | **Application** | **Ref** |
| AMSCs | - | Beneficial effect on the growth and osteogenic differentiation of BMSCs | Rat calvarial bone defect models | Bone defect | (Kyung Kim et al., 2021) |
| IPSCs | - | Promote cartilage defect repair | Rabbit articular cartilage defect models | Cartilage defects | (Liu et al., 2017) |
| HESC-MSCs | - | Promote TMJ repair and regeneration in OA | Rat TMJ-OA models | TMJ-OA | (Zhang et al., 2019a) |
| AMSCs | - | Ameliorate hypoxia/serum deprivation-induced osteocyte apoptosis and osteocyte-mediated osteoclastogenesis | Osteocyte-like cell line MLO-Y4 | Age-related bone disease | (Ren et al., 2019) |
| AMSCs | - | Attenuate cytotoxicity and apoptosis of TNF-α-induced MC3T3-E1 cells | MC3T3-E1 cells were treated with different doses of tumor necrosis factor-alpha (TNF-α) | OP | (Wang et al., 2021c) |
| AMSCs |  | Alleviate diabetic OP in rats through suppressing NLRP3 inflammasome activation in osteoclasts | Diabetic OP rat models | Diabetic OP | (Zhang et al., 2021d) |
| AMSCs | - | Promote the proliferation and osteogenic differentiation in human primary osteoblastic cells | AMSCs were pre-conditioned with TNF-α for 3 days, which mimics the acute inflammatory phase upon bone injury | Bone regeneration | (Lu et al., 2017) |
| AMSCs | - | Induce osteogenic differentiation of hAMSCs, promote the proliferation and  migration of hAMSCs | hAMSCs | Bone defect | (Zhu et al., 2021) |
| AMSCs | - | Enhance bone regeneration through its osteoinductive effects and capacities of promoting MSC migration and homing in the newly-formed bone tissue | Mouse calvarial defect models | Bone defect | (Li et al., 2018) |
| AMSCs | - | Facilitate cartilage injury repair and improve osteoarthritis | Rat cartilage defect models | OA | (Li et al., 2021a) |
| IPSCs | - | Enhance angiogenesis and osteogenesis | Ovariectomized rat models | OP | (Qi et al., 2016) |
| HiPSC-MSC-Exos | - | Enhance the proliferation, migration, and osteogenic differentiation of BMSCs | Rat calvarial bone defect models | Bone defect | (Zhang et al., 2016) |
| IPSCs | - | Promote proliferation and migration of human chondrocytes | Mouse OA models | OA | (Zhu et al., 2017) |
| HESCs |  | Promote osteochondral regeneration | Rat osteochondral defect models | Cartilage repair | (Zhang, 2016 #217) |
| HESCs | - | Promote osteochondral regeneration | Rat osteochondral defect models | Osteochondral defect | (Zhang et al., 2018a) |
| Human gingival stem cells | - | Exhibit osteogenic inductivity and improve bone healing | Rat cortical calvaria bone tissue damage models | Bone defect | (Diomede et al., 2018) |
|  |  |  |  |  |  |
| **Sources** | **MiRs** | **Functions** | **Model system** | **Application** | **Ref** |
| Human dental pulp stem cells | - | Facilitate osteogenic differentiation of BMSCs and bone healing | Mouse calvarial bone defect models | Bone defect | (Swanson et al., 2020) |
| Human dental pulp stem cells | - | Promoted osteogenic differentiation of  homotypic dental pulp stem cells | Dental pulp stem cells | Bone regeneration | (Xie et al., 2020) |
| Periodontal ligament stem cells | - | Improve proliferation, migration, and osteogenic differentiation of BMSCs | Rat alveolar bone defect models | Bone defect | (Yu et al., 2021) |
| Human perivascular stem cells | - | Stimulate bone repair, and did so via stimulation of skeletal cell proliferation, migration, and osteodifferentiation. | Mouse calvarial defect models | Bone fracture | (Xu et al., 2019) |
| Amniotic fluid stem cells | - | Promote the polarization of macrophages and cartilage repair | Rat OA models | OA | (Zavatti et al., 2020) |
| Human synovial  mesenchymal stem cells | - | Inhibit extracellular matrix degradation and prevent osteoarthritis | Mouse OA models | OA | (Duan et al., 2021) |
| Antler stem cells | - | Alleviate MSCs senescence and OA | Mouse OA models | OA | (Lei et al., 2021) |

**Table S2.** Bone/cartilage cell-derived exosomes

| **Sources** | **miRs** | **Functions** | **Model system** | **Application** | **Ref** |
| --- | --- | --- | --- | --- | --- |
| Osteoclasts | MiR-214 | Inhibit osteoblast activity | Ovariectomized mouse models | OP | (Sun et al., 2016) |
| Osteoclasts | MiR-214-3p | Inhibit osteoblastic bone formation | Ovariectomized mouse models | OP | (Li et al., 2016) |
| Osteoblasts | MiR-503-3p | Inhibit the osteoclast differentiation via miR-503-3p/Hpse axis | Osteoclast progenitor cells | Bone homeostasis | (Wang et al., 2021b) |
| Osteoblasts | - | Potential to affect osteogenesis | Mouse osteoblasts cell line Mc3t3 | Bone disorders and metastases | (Ge et al., 2015) |
| Osteoblasts | - | Close relationship in bone diseases | Mouse osteoblasts cell line Mc3t3 | Bone disorders and metastases | (Ge et al., 2017) |
| Osteoblasts | - | Reverse OVX-induced osteoporosis | Ovariectomized rat models | OP | (Sadat-Ali et al., 2021) |
| Osteoblasts | - | Promote BMSCs differentiation to osteoblasts | BMSCs | Bone tissue engineering | (Cui et al., 2016) |
| Osteoblasts | - | Promote BMSCs osteogenic differentiation | BMSCs | Bone repair | (Man et al., 2021) |
| Osteoblasts | - | Decrease BMSCs viability and alkaline phosphatase gene expression | BMSCs | OA and OP | (Niedermair et al., 2020) |
| CPCs | - | Enhance osteogenic induction and vascular remodeling | Rat radial defect models | Bone defect | (Zha et al., 2021) |
| CPCs | MiR-221-3p | Stimulate chondrocyte proliferation and migration | Mouse OA models | OA | (Wang et al., 2020b) |
| Chondrocytes | - | Facilitate subcutaneous stable ectopic chondrogenesis of CPCs | Nude mouse models | Cartilage regeneration | (Chen et al., 2018) |

**Table S3.** Monocyte and macrophage-derived exosomes

| **Sources** | **miRs** | **Functions** | **Model system** | **Application** | **Ref** |
| --- | --- | --- | --- | --- | --- |
| Macrophages | - | Promote angiogenesis | Rat fracture nonunion models | Bone fracture | (Wang et al., 2021a) |
| Macrophages | MiR-155,  miR-378a | M1 macrophage EVs-enriched miR-155 reduces MSC osteogenic differentiation, M2 macrophage EVs-enriched miR-378a increases MSC osteoinductive gene expression | Rat calvarial bone defect models | Bone defect | (Kang et al., 2020) |
| Monocytes | - | Stimulate the osteogenic gene expression of MSCs | BMSCs | Bone regeneration | (Ekstrom et al., 2013) |
| Macrophages | - | M1 macrophages-derived exosomes supports BMSCs proliferation, osteogenic and adipogenic differentiation. | BMSCs | Tissue  regeneration | (Xia et al., 2020) |
| Macrophages | MiR-690 | M2 macrophages facilitate osteogenesis and reduce adipogenesis of BMSCs | BMSCs | Bone loss diseases | (Li et al., 2021b) |
| Macrophages | MiR-5106 | M2 Macrophagy-derived exosomal miRNA-5106 induces BMSCs towards osteoblasts | Mouse femoral fracture models | Bone fracture | (Xiong et al., 2020) |
| Macrophages | MiR-144-5p | Impair bone fracture healing in type 2 diabetes | Rat femoral fracture models | Bone fracture | (Zhang et al., 2021b) |
| Macrophages | LncRNA MM2P | Promote chondrocyte differentiation and functions | Mouse primary chondrocytes | OA | (Bai et al., 2020) |
| Macrophages | - | Facilitate BMSCs osteogenic differentiation | BMSCs | Bone fracture | (Wei et al., 2019) |
| Macrophages | - | Promote osteogenic differentiation and mineralization of MC3T3-E1 cells | MC3T3-E1 cells | Osseointegration | (Zhang et al., 2021e) |
| Macrophages | - | Promote biomimetic mineralized collagen-mediated endogenous bone regeneration | Rat critical-sized mandible defect models | Bone defect | (Liu et al., 2020a) |

**Table S4.** Serum and plasma-derived exosomes

| **Sources** | **miRs** | **Functions** | **Model system** | **Application** | **Ref** |
| --- | --- | --- | --- | --- | --- |
| Serum | - | Involve in suppressing the integrin-mediated mechanosensation and activation of osteoblastic cells, trigger the differentiation and resorption of osteoclasts | Elderly OP patients’ serum-derived exosomes | OP | (Xie et al., 2018) |
| Plasma | - | Promote the proliferation and differentiation of osteoblasts | Mouse tibia fracture with traumatic brain injury models | Bone fracture | (Yang et al., 2021) |
| Plasma | MiR-642a-3p | Contribute to the prediction and diagnosis of early postmenopausal osteoporosis | Exosomes in early postmenopausal women | OP | (Kong et al., 2021) |
| Plasma | TRF‐25, tRF‐38, tRF‐18 | Diagnostic biomarkers for osteoporosis detection | OP patients | OP | (Zhang et al., 2018b) |
| Plasma | - | Provide references for further investigations into the pathological mechanisms of OP | Participants from the PLAGH Hip Fracture Database. | OP | (Chen et al., 2020b) |
| Serum | Hsa_circ_0006859 | A potential biomarker for OP and enhances adipogenic versus osteogenic differentiation in BMSCs | BMSCs | OP | (Zhi et al., 2021) |
| Serum | LncRNAs | Potential diagnostic markers and therapeutic modules for OP | OP patients | OP | (Teng et al., 2020) |
| Serum | - | Serum derived-exosomes from young rats improve the reduced osteogenic differentiation of BMSCs in aged rats with OP after fatigue loading | Ovariectomized rats after fatigue loading models | OP | (Xun et al., 2021) |
| Plasma | - | Radiation can affect bone metabolism and regeneration. | Rabbit radiation models | Radiation bone injury | (Du et al., 2021) |

**Table S5.** Other exosomes

| **Sources** | **MiRs** | **Functions** | **Model system** | **Application** | **Ref** |
| --- | --- | --- | --- | --- | --- |
| Rat sinus mucosa and periosteum | - | Enhance the proliferation, migration and osteogenic differentiation of BMSCs, accelerate bone formation | Rat femoral fracture models | Guided bone regeneration | (Sun et al., 2019) |
| ECs | MiR-155 | Alter macrophages morphology and inhibit osteoclast activity | Ovariectomized mouse models | OP | (Song et al., 2019) |
| EPCs | LncRNA MALAT1 | Promote bone repair by enhancing recruitment and differentiation of osteoclast precursors through LncRNA‐MALAT1 | Ovariectomized mouse models | Bone fracture | (Cui et al., 2019) |
| EPCs | - | Accelerate bone regeneration during distraction osteogenesis by stimulating angiogenesis | Rat unilateral tibial distraction osteogenesis models | Distraction osteogenesis | (Jia et al., 2019) |
| FLSs | LncRNA H19 | Promote chondrocyte proliferation and migration and inhibit matrix degradation in OA | Chondrocytes | OA | (Tan et al., 2020) |
| FLSs | MiR-486-5p | Induce osteoblast differentiation | Mouse RA models | RA | (Chen et al., 2020a) |
| Neurons | - | Target osteoprogenitors in bone to stimulate bone formation | Rat critical-sized calvarial defects models | Bone healing and  bone nonunion | (Xia et al., 2021) |

References

Bai, J., Zhang, Y., Zheng, X., Huang, M., Cheng, W., Shan, H., et al. (2020). LncRNA MM2P-induced, exosome-mediated transfer of Sox9 from monocyte-derived cells modulates primary chondrocytes. *Cell Death Dis* 11(9)**,** 763. doi: 10.1038/s41419-020-02945-5.

Chen, J., Liu, M., Luo, X., Peng, L., Zhao, Z., He, C., et al. (2020a). Exosomal miRNA-486-5p derived from rheumatoid arthritis fibroblast-like synoviocytes induces osteoblast differentiation through the Tob1/BMP/Smad pathway. *Biomater Sci* 8(12)**,** 3430-3442. doi: 10.1039/c9bm01761e.

Chen, M., Li, Y., Lv, H., Yin, P., Zhang, L., and Tang, P. (2020b). Quantitative proteomics and reverse engineer analysis identified plasma exosome derived protein markers related to osteoporosis. *J Proteomics* 228**,** 103940. doi: 10.1016/j.jprot.2020.103940.

Chen, P., Zheng, L., Wang, Y., Tao, M., Xie, Z., Xia, C., et al. (2019). Desktop-stereolithography 3D printing of a radially oriented extracellular matrix/mesenchymal stem cell exosome bioink for osteochondral defect regeneration. *Theranostics* 9(9)**,** 2439-2459. doi: 10.7150/thno.31017.

Chen, X., Shi, Y., Xue, P., Ma, X., Li, J., and Zhang, J. (2020c). Mesenchymal stem cell-derived exosomal microRNA-136-5p inhibits chondrocyte degeneration in traumatic osteoarthritis by targeting ELF3. *Arthritis Res Ther* 22(1)**,** 256. doi: 10.1186/s13075-020-02325-6.

Chen, Y., Xue, K., Zhang, X., Zheng, Z., and Liu, K. (2018). Exosomes derived from mature chondrocytes facilitate subcutaneous stable ectopic chondrogenesis of cartilage progenitor cells. *Stem Cell Res Ther* 9(1)**,** 318. doi: 10.1186/s13287-018-1047-2.

Cui, Y., Fu, S., Sun, D., Xing, J., Hou, T., and Wu, X. (2019). EPC-derived exosomes promote osteoclastogenesis through LncRNA-MALAT1. *J Cell Mol Med* 23(6)**,** 3843-3854. doi: 10.1111/jcmm.14228.

Cui, Y., Luan, J., Li, H., Zhou, X., and Han, J. (2016). Exosomes derived from mineralizing osteoblasts promote ST2 cell osteogenic differentiation by alteration of microRNA expression. *FEBS Lett* 590(1)**,** 185-192. doi: 10.1002/1873-3468.12024.

Diomede, F., Gugliandolo, A., Cardelli, P., Merciaro, I., Ettorre, V., Traini, T., et al. (2018). Three-dimensional printed PLA scaffold and human gingival stem cell-derived extracellular vesicles: a new tool for bone defect repair. *Stem Cell Res Ther* 9(1)**,** 104. doi: 10.1186/s13287-018-0850-0.

Du, Y., Tang, H., Gu, X., Shi, Y., Gong, P., and Yao, Y. (2021). Radiation Can Regulate the Expression of miRNAs Associated with Osteogenesis and Oxidation in Exosomes from Peripheral Blood Plasma. *Oxid Med Cell Longev* 2021**,** 6646323. doi: 10.1155/2021/6646323.

Duan, A., Shen, K., Li, B., Li, C., Zhou, H., Kong, R., et al. (2021). Extracellular vesicles derived from LPS-preconditioned human synovial mesenchymal stem cells inhibit extracellular matrix degradation and prevent osteoarthritis of the knee in a mouse model. *Stem Cell Res Ther* 12(1)**,** 427. doi: 10.1186/s13287-021-02507-2.

Ekstrom, K., Omar, O., Graneli, C., Wang, X., Vazirisani, F., and Thomsen, P. (2013). Monocyte exosomes stimulate the osteogenic gene expression of mesenchymal stem cells. *PLoS One* 8(9)**,** e75227. doi: 10.1371/journal.pone.0075227.

Fan, J., Lee, C.S., Kim, S., Chen, C., Aghaloo, T., and Lee, M. (2020). Generation of Small RNA-Modulated Exosome Mimetics for Bone Regeneration. *ACS Nano* 14(9)**,** 11973-11984. doi: 10.1021/acsnano.0c05122.

Furuta, T., Miyaki, S., Ishitobi, H., Ogura, T., Kato, Y., Kamei, N., et al. (2016). Mesenchymal Stem Cell-Derived Exosomes Promote Fracture Healing in a Mouse Model. *Stem Cells Transl Med* 5(12)**,** 1620-1630. doi: 10.5966/sctm.2015-0285.

Ge, M., Ke, R., Cai, T., Yang, J., and Mu, X. (2015). Identification and proteomic analysis of osteoblast-derived exosomes. *Biochem Biophys Res Commun* 467(1)**,** 27-32. doi: 10.1016/j.bbrc.2015.09.135.

Ge, M., Wu, Y., Ke, R., Cai, T., Yang, J., and Mu, X. (2017). Value of Osteoblast-Derived Exosomes in Bone Diseases. *J Craniofac Surg* 28(4)**,** 866-870. doi: 10.1097/SCS.0000000000003463.

Hassan, M.Q., Maeda, Y., Taipaleenmaki, H., Zhang, W., Jafferji, M., Gordon, J.A., et al. (2012). miR-218 directs a Wnt signaling circuit to promote differentiation of osteoblasts and osteomimicry of metastatic cancer cells. *J Biol Chem* 287(50)**,** 42084-42092. doi: 10.1074/jbc.M112.377515.

He, L., Chen, Y., Ke, Z., Pang, M., Yang, B., Feng, F., et al. (2020a). Exosomes derived from miRNA-210 overexpressing bone marrow mesenchymal stem cells protect lipopolysaccharide induced chondrocytes injury via the NF-kappaB pathway. *Gene* 751**,** 144764. doi: 10.1016/j.gene.2020.144764.

He, L., He, T., Xing, J., Zhou, Q., Fan, L., Liu, C., et al. (2020b). Bone marrow mesenchymal stem cell-derived exosomes protect cartilage damage and relieve knee osteoarthritis pain in a rat model of osteoarthritis. *Stem Cell Res Ther* 11(1)**,** 276. doi: 10.1186/s13287-020-01781-w.

Hingert, D., Ekstrom, K., Aldridge, J., Crescitelli, R., and Brisby, H. (2020). Extracellular vesicles from human mesenchymal stem cells expedite chondrogenesis in 3D human degenerative disc cell cultures. *Stem Cell Res Ther* 11(1)**,** 323. doi: 10.1186/s13287-020-01832-2.

Hu, H., Dong, L., Bu, Z., Shen, Y., Luo, J., Zhang, H., et al. (2020a). miR-23a-3p-abundant small extracellular vesicles released from Gelma/nanoclay hydrogel for cartilage regeneration. *J Extracell Vesicles* 9(1)**,** 1778883. doi: 10.1080/20013078.2020.1778883.

Hu, Y., Li, X., Zhang, Q., Gu, Z., Luo, Y., Guo, J., et al. (2021). Exosome-guided bone targeted delivery of Antagomir-188 as an anabolic therapy for bone loss. *Bioact Mater* 6(9)**,** 2905-2913. doi: 10.1016/j.bioactmat.2021.02.014.

Hu, Y., Zhang, Y., Ni, C.Y., Chen, C.Y., Rao, S.S., Yin, H., et al. (2020b). Human umbilical cord mesenchymal stromal cells-derived extracellular vesicles exert potent bone protective effects by CLEC11A-mediated regulation of bone metabolism. *Theranostics* 10(5)**,** 2293-2308. doi: 10.7150/thno.39238.

Huang, C.-C., Kang, M., Lu, Y., Shirazi, S., Diaz, J.I., Cooper, L.F., et al. (2020). Functionally engineered extracellular vesicles improve bone regeneration. *Acta Biomaterialia* 109**,** 182-194. doi: 10.1016/j.actbio.2020.04.017.

Huang, C.C., Kang, M., Shirazi, S., Lu, Y., Cooper, L.F., Gajendrareddy, P., et al. (2021a). 3D Encapsulation and tethering of functionally engineered extracellular vesicles to hydrogels. *Acta Biomater* 126**,** 199-210. doi: 10.1016/j.actbio.2021.03.030.

Huang, Y., Xu, Y., Feng, S., He, P., Sheng, B., and Ni, J. (2021b). miR-19b enhances osteogenic differentiation of mesenchymal stem cells and promotes fracture healing through the WWP1/Smurf2-mediated KLF5/beta-catenin signaling pathway. *Exp Mol Med* 53(5)**,** 973-985. doi: 10.1038/s12276-021-00631-w.

Huang, Y., Zhang, X., Zhan, J., Yan, Z., Chen, D., Xue, X., et al. (2021c). Bone marrow mesenchymal stem cell-derived exosomal miR-206 promotes osteoblast proliferation and differentiation in osteoarthritis by reducing Elf3. *J Cell Mol Med* 25(16)**,** 7734-7745. doi: 10.1111/jcmm.16654.

Jia, Y., Qiu, S., Xu, J., Kang, Q., and Chai, Y. (2020). Exosomes Secreted by Young Mesenchymal Stem Cells Promote New Bone Formation During Distraction Osteogenesis in Older Rats. *Calcif Tissue Int* 106(5)**,** 509-517. doi: 10.1007/s00223-019-00656-4.

Jia, Y., Zhu, Y., Qiu, S., Xu, J., and Chai, Y. (2019). Exosomes secreted by endothelial progenitor cells accelerate bone regeneration during distraction osteogenesis by stimulating angiogenesis. *Stem Cell Res Ther* 10(1)**,** 12. doi: 10.1186/s13287-018-1115-7.

Jiang, S., Tian, G., Yang, Z., Gao, X., Wang, F., Li, J., et al. (2021). Enhancement of acellular cartilage matrix scaffold by Wharton's jelly mesenchymal stem cell-derived exosomes to promote osteochondral regeneration. *Bioact Mater* 6(9)**,** 2711-2728. doi: 10.1016/j.bioactmat.2021.01.031.

Jin, Z., Ren, J., and Qi, S. (2020). Human bone mesenchymal stem cells-derived exosomes overexpressing microRNA-26a-5p alleviate osteoarthritis via down-regulation of PTGS2. *Int Immunopharmacol* 78**,** 105946. doi: 10.1016/j.intimp.2019.105946.

Kang, M., Huang, C.C., Lu, Y., Shirazi, S., Gajendrareddy, P., Ravindran, S., et al. (2020). Bone regeneration is mediated by macrophage extracellular vesicles. *Bone* 141**,** 115627. doi: 10.1016/j.bone.2020.115627.

Kong, D., Chen, T., Zheng, X., Yang, T., Zhang, Y., and Shao, J. (2021). Comparative profile of exosomal microRNAs in postmenopausal women with various bone mineral densities by small RNA sequencing. *Genomics* 113(3)**,** 1514-1521. doi: 10.1016/j.ygeno.2021.03.028.

Kuang, M.J., Huang, Y., Zhao, X.G., Zhang, R., Ma, J.X., Wang, D.C., et al. (2019). Exosomes derived from Wharton's jelly of human umbilical cord mesenchymal stem cells reduce osteocyte apoptosis in glucocorticoid-induced osteonecrosis of the femoral head in rats via the miR-21-PTEN-AKT signalling pathway. *Int J Biol Sci* 15(9)**,** 1861-1871. doi: 10.7150/ijbs.32262.

Kyung Kim, D., Lee, S., Kim, M., Jeong, Y., and Lee, S. (2021). Exosome-coated silk fibroin 3D-scaffold for inducing osteogenic differentiation of bone marrow derived mesenchymal stem cells. *Chemical Engineering Journal* 406. doi: 10.1016/j.cej.2020.127080.

Lei, J., Jiang, X., Li, W., Ren, J., Wang, D., Ji, Z., et al. (2021). Exosomes from antler stem cells alleviate mesenchymal stem cell senescence and osteoarthritis. *Protein Cell*. doi: 10.1007/s13238-021-00860-9.

Li, D., Liu, J., Guo, B., Liang, C., Dang, L., Lu, C., et al. (2016). Osteoclast-derived exosomal miR-214-3p inhibits osteoblastic bone formation. *Nat Commun* 7**,** 10872. doi: 10.1038/ncomms10872.

Li, S., Liu, J., Liu, S., Jiao, W., and Wang, X. (2021a). Chitosan oligosaccharides packaged into rat adipose mesenchymal stem cells-derived extracellular vesicles facilitating cartilage injury repair and alleviating osteoarthritis. *J Nanobiotechnology* 19(1)**,** 343. doi: 10.1186/s12951-021-01086-x.

Li, W., Liu, Y., Zhang, P., Tang, Y., Zhou, M., Jiang, W., et al. (2018). Tissue-Engineered Bone Immobilized with Human Adipose Stem Cells-Derived Exosomes Promotes Bone Regeneration. *ACS Appl Mater Interfaces* 10(6)**,** 5240-5254. doi: 10.1021/acsami.7b17620.

Li, Y., Wang, J., Ma, Y., Du, W., Feng, H., Feng, K., et al. (2020). MicroRNA-15b shuttled by bone marrow mesenchymal stem cell-derived extracellular vesicles binds to WWP1 and promotes osteogenic differentiation. *Arthritis Res Ther* 22(1)**,** 269. doi: 10.1186/s13075-020-02316-7.

Li, Z., Wang, Y., Li, S., and Li, Y. (2021b). Exosomes Derived From M2 Macrophages Facilitate Osteogenesis and Reduce Adipogenesis of BMSCs. *Front Endocrinol (Lausanne)* 12**,** 680328. doi: 10.3389/fendo.2021.680328.

Liang, B., Liang, J.M., Ding, J.N., Xu, J., Xu, J.G., and Chai, Y.M. (2019). Dimethyloxaloylglycine-stimulated human bone marrow mesenchymal stem cell-derived exosomes enhance bone regeneration through angiogenesis by targeting the AKT/mTOR pathway. *Stem Cell Res Ther* 10(1)**,** 335. doi: 10.1186/s13287-019-1410-y.

Liang, Z., Yang, L., and Lv, Y. (2021). Exosome derived from mesenchymal stem cells mediates hypoxia-specific BMP2 gene delivery and enhances bone regeneration. *Chemical Engineering Journal* 422. doi: 10.1016/j.cej.2021.130084.

Liao, Q., Li, B.J., Li, Y., Xiao, Y., Zeng, H., Liu, J.M., et al. (2021). Low-intensity pulsed ultrasound promotes osteoarthritic cartilage regeneration by BMSC-derived exosomes via modulating the NF-kappaB signaling pathway. *Int Immunopharmacol* 97**,** 107824. doi: 10.1016/j.intimp.2021.107824.

Liao, W., Ning, Y., Xu, H.J., Zou, W.Z., Hu, J., Liu, X.Z., et al. (2019). BMSC-derived exosomes carrying microRNA-122-5p promote proliferation of osteoblasts in osteonecrosis of the femoral head. *Clin Sci (Lond)* 133(18)**,** 1955-1975. doi: 10.1042/CS20181064.

Liu, A., Jin, S., Fu, C., Cui, S., Zhang, T., Zhu, L., et al. (2020a). Macrophage-derived small extracellular vesicles promote biomimetic mineralized collagen-mediated endogenous bone regeneration. *Int J Oral Sci* 12(1)**,** 33. doi: 10.1038/s41368-020-00100-6.

Liu, A., Lin, D., Zhao, H., Chen, L., Cai, B., Lin, K., et al. (2021a). Optimized BMSC-derived osteoinductive exosomes immobilized in hierarchical scaffold via lyophilization for bone repair through Bmpr2/Acvr2b competitive receptor-activated Smad pathway. *Biomaterials* 272**,** 120718. doi: 10.1016/j.biomaterials.2021.120718.

Liu, C., Li, Y., Yang, Z.J., Zhou, Z.Y., Lou, Z.H., and Zhang, Q.Q. (2020b). Kartogenin enhances the therapeutic effect of bone marrow mesenchymal stem cells derived exosomes in cartilage repair. *Nanomedicine* 15**,** 273-288. doi: 10.2217/nnm-2019-0208.

Liu, L., Yu, F., Li, L., Zhou, L., Zhou, T., Xu, Y., et al. (2021b). Bone marrow stromal cells stimulated by strontium-substituted calcium silicate ceramics: release of exosomal miR-146a regulates osteogenesis and angiogenesis. *Acta Biomater* 119**,** 444-457. doi: 10.1016/j.actbio.2020.10.038.

Liu, W., Li, L., Rong, Y., Qian, D., Chen, J., Zhou, Z., et al. (2020c). Hypoxic mesenchymal stem cell-derived exosomes promote bone fracture healing by the transfer of miR-126. *Acta Biomater* 103**,** 196-212. doi: 10.1016/j.actbio.2019.12.020.

Liu, X., Yang, Y., Li, Y., Niu, X., Zhao, B., Wang, Y., et al. (2017). Integration of stem cell-derived exosomes with in situ hydrogel glue as a promising tissue patch for articular cartilage regeneration. *Nanoscale* 9(13)**,** 4430-4438. doi: 10.1039/c7nr00352h.

Lu, Z., Chen, Y., Dunstan, C., Roohani-Esfahani, S., and Zreiqat, H. (2017). Priming Adipose Stem Cells with Tumor Necrosis Factor-Alpha Preconditioning Potentiates Their Exosome Efficacy for Bone Regeneration. *Tissue Eng Part A* 23(21-22)**,** 1212-1220. doi: 10.1089/ten.tea.2016.0548.

Luo, Z.W., Li, F.X., Liu, Y.W., Rao, S.S., Yin, H., Huang, J., et al. (2019). Aptamer-functionalized exosomes from bone marrow stromal cells target bone to promote bone regeneration. *Nanoscale* 11(43)**,** 20884-20892. doi: 10.1039/c9nr02791b.

Man, K., Brunet, M.Y., Fernandez-Rhodes, M., Williams, S., Heaney, L.M., Gethings, L.A., et al. (2021). Epigenetic reprogramming enhances the therapeutic efficacy of osteoblast-derived extracellular vesicles to promote human bone marrow stem cell osteogenic differentiation. *J Extracell Vesicles* 10(9)**,** e12118. doi: 10.1002/jev2.12118.

Mao, G., Xu, Y., Long, D., Sun, H., Li, H., Xin, R., et al. (2021). Exosome-transported circRNA_0001236 enhances chondrogenesis and suppress cartilage degradation via the miR-3677-3p/Sox9 axis. *Stem Cell Res Ther* 12(1)**,** 389. doi: 10.1186/s13287-021-02431-5.

Niedermair, T., Lukas, C., Li, S., Stockl, S., Craiovan, B., Brochhausen, C., et al. (2020). Influence of Extracellular Vesicles Isolated From Osteoblasts of Patients With Cox-Arthrosis and/or Osteoporosis on Metabolism and Osteogenic Differentiation of BMSCs. *Front Bioeng Biotechnol* 8**,** 615520. doi: 10.3389/fbioe.2020.615520.

Qi, X., Zhang, J., Yuan, H., Xu, Z., Li, Q., Niu, X., et al. (2016). Exosomes Secreted by Human-Induced Pluripotent Stem Cell-Derived Mesenchymal Stem Cells Repair Critical-Sized Bone Defects through Enhanced Angiogenesis and Osteogenesis in Osteoporotic Rats. *Int J Biol Sci* 12(7)**,** 836-849. doi: 10.7150/ijbs.14809.

Ren, L., Song, Z.J., Cai, Q.W., Chen, R.X., Zou, Y., Fu, Q., et al. (2019). Adipose mesenchymal stem cell-derived exosomes ameliorate hypoxia/serum deprivation-induced osteocyte apoptosis and osteocyte-mediated osteoclastogenesis in vitro. *Biochem Biophys Res Commun* 508(1)**,** 138-144. doi: 10.1016/j.bbrc.2018.11.109.

Sadat-Ali, M., Al-Dakheel, D., Al-Turki, H., and Acharya, S. (2021). Efficacy of autologous bone marrow derived Mesenchymal stem cells (MSCs), osteoblasts and osteoblasts derived exosome in the reversal of ovariectomy (OVX) induced osteoporosis in rabbit model. *AM J Transl Res* 13**,** 6175-6181.

Song, H., Li, X., Zhao, Z., Qian, J., Wang, Y., Cui, J., et al. (2019). Reversal of Osteoporotic Activity by Endothelial Cell-Secreted Bone Targeting and Biocompatible Exosomes. *Nano Lett* 19(5)**,** 3040-3048. doi: 10.1021/acs.nanolett.9b00287.

Sun, R., Xu, S., and Wang, Z. (2019). Rat sinus mucosa- and periosteum-derived exosomes accelerate osteogenesis. *J Cell Physiol* 234(12)**,** 21947-21961. doi: 10.1002/jcp.28758.

Sun, W., Zhao, C., Li, Y., Wang, L., Nie, G., Peng, J., et al. (2016). Osteoclast-derived microRNA-containing exosomes selectively inhibit osteoblast activity. *Cell Discov* 2**,** 16015. doi: 10.1038/celldisc.2016.15.

Swanson, W.B., Zhang, Z., Xiu, K., Gong, T., Eberle, M., Wang, Z., et al. (2020). Scaffolds with controlled release of pro-mineralization exosomes to promote craniofacial bone healing without cell transplantation. *Acta Biomater* 118**,** 215-232. doi: 10.1016/j.actbio.2020.09.052.

Tan, F., Wang, D., and Yuan, Z. (2020). The Fibroblast-Like Synoviocyte Derived Exosomal Long Non-coding RNA H19 Alleviates Osteoarthritis Progression Through the miR-106b-5p/TIMP2 Axis. *Inflammation* 43(4)**,** 1498-1509. doi: 10.1007/s10753-020-01227-8.

Teng, Z., Zhu, Y., Zhang, X., Teng, Y., and Lu, S. (2020). Osteoporosis Is Characterized by Altered Expression of Exosomal Long Non-coding RNAs. *Front Genet* 11**,** 566959. doi: 10.3389/fgene.2020.566959.

Vonk, L.A., van Dooremalen, S.F.J., Liv, N., Klumperman, J., Coffer, P.J., Saris, D.B.F., et al. (2018). Mesenchymal Stromal/stem Cell-derived Extracellular Vesicles Promote Human Cartilage Regeneration In Vitro. *Theranostics* 8(4)**,** 906-920. doi: 10.7150/thno.20746.

Wang, D., Wang, J., Zhou, J., and Zheng, X. (2021a). The Role of Adenosine Receptor A2A in the Regulation of Macrophage Exosomes and Vascular Endothelial Cells During Bone Healing. *J Inflamm Res* 14**,** 4001-4017. doi: 10.2147/JIR.S324232.

Wang, L., Wang, J., Zhou, X., Sun, J., Zhu, B., Duan, C., et al. (2020a). A New Self-Healing Hydrogel Containing hucMSC-Derived Exosomes Promotes Bone Regeneration. *Front Bioeng Biotechnol* 8**,** 564731. doi: 10.3389/fbioe.2020.564731.

Wang, Q., Shen, X., Chen, Y., Chen, J., and Li, Y. (2021b). Osteoblasts-derived exosomes regulate osteoclast differentiation through miR-503-3p/Hpse axis. *Acta Histochem* 123(7)**,** 151790. doi: 10.1016/j.acthis.2021.151790.

Wang, R., Jiang, W., Zhang, L., Xie, S., Zhang, S., Yuan, S., et al. (2020b). Intra-articular delivery of extracellular vesicles secreted by chondrogenic progenitor cells from MRL/MpJ superhealer mice enhances articular cartilage repair in a mouse injury model. *Stem Cell Res Ther* 11(1)**,** 93. doi: 10.1186/s13287-020-01594-x.

Wang, S., Jia, J., and Chen, C.-h. (2021c). LncRNA-KCNQ1OT1: A Potential Target in Exosomes Derived From ADSCs for The Treatment of Osteoporosis. *Stem Cells Int*. doi: 10.21203/rs.3.rs-576971/v1.

Wei, F., Li, M., Crawford, R., Zhou, Y., and Xiao, Y. (2019). Exosome-integrated titanium oxide nanotubes for targeted bone regeneration. *Acta Biomater* 86**,** 480-492. doi: 10.1016/j.actbio.2019.01.006.

Wong, K.L., Zhang, S., Wang, M., Ren, X., Afizah, H., Lai, R.C., et al. (2020). Intra-Articular Injections of Mesenchymal Stem Cell Exosomes and Hyaluronic Acid Improve Structural and Mechanical Properties of Repaired Cartilage in a Rabbit Model. *Arthroscopy* 36(8)**,** 2215-2228 e2212. doi: 10.1016/j.arthro.2020.03.031.

Xia, W., Xie, J., Cai, Z., Liu, X., Wen, J., Cui, Z.K., et al. (2021). Damaged brain accelerates bone healing by releasing small extracellular vesicles that target osteoprogenitors. *Nat Commun* 12(1)**,** 6043. doi: 10.1038/s41467-021-26302-y.

Xia, Y., He, X.T., Xu, X.Y., Tian, B.M., An, Y., and Chen, F.M. (2020). Exosomes derived from M0, M1 and M2 macrophages exert distinct influences on the proliferation and differentiation of mesenchymal stem cells. *PeerJ* 8**,** e8970. doi: 10.7717/peerj.8970.

Xie, L., Guan, Z., Zhang, M., Lyu, S., Thuaksuban, N., Kamolmattayakul, S., et al. (2020). Exosomal circLPAR1 Promoted Osteogenic Differentiation of Homotypic Dental Pulp Stem Cells by Competitively Binding to hsa-miR-31. *Biomed Res Int* 2020**,** 6319395. doi: 10.1155/2020/6319395.

Xie, Y., Gao, Y., Zhang, L., Chen, Y., Ge, W., and Tang, P. (2018). Involvement of serum-derived exosomes of elderly patients with bone loss in failure of bone remodeling via alteration of exosomal bone-related proteins. *Aging Cell* 17(3)**,** e12758. doi: 10.1111/acel.12758.

Xiong, Y., Chen, L., Yan, C., Zhou, W., Yu, T., Sun, Y., et al. (2020). M2 Macrophagy-derived exosomal miRNA-5106 induces bone mesenchymal stem cells towards osteoblastic fate by targeting salt-inducible kinase 2 and 3. *J Nanobiotechnology* 18(1)**,** 66. doi: 10.1186/s12951-020-00622-5.

Xu, J., Wang, Y., Hsu, C.Y., Gao, Y., Meyers, C.A., Chang, L., et al. (2019). Human perivascular stem cell-derived extracellular vesicles mediate bone repair. *Elife* 8. doi: 10.7554/eLife.48191.

Xu, R., Shen, X., Si, Y., Fu, Y., Zhu, W., Xiao, T., et al. (2018). MicroRNA-31a-5p from aging BMSCs links bone formation and resorption in the aged bone marrow microenvironment. *Aging Cell* 17(4)**,** e12794. doi: 10.1111/acel.12794.

Xu, S., and Wang, Z. (2017). Bone marrow mesenchymal stem cell-derived exosomes enhance osteoclastogenesis during alveolar bone deterioration in rats. *RSC Advances* 7(34)**,** 21153-21163. doi: 10.1039/c6ra27931g.

Xu, T., Luo, Y., Wang, J., Zhang, N., Gu, C., Li, L., et al. (2020). Exosomal miRNA-128-3p from mesenchymal stem cells of aged rats regulates osteogenesis and bone fracture healing by targeting Smad5. *J Nanobiotechnology* 18(1)**,** 47. doi: 10.1186/s12951-020-00601-w.

Xun, J., Li, C., Liu, M., Mei, Y., Zhou, Q., Wu, B., et al. (2021). Serum exosomes from young rats improve the reduced osteogenic differentiation of BMSCs in aged rats with osteoporosis after fatigue loading in vivo. *Stem Cell Res Ther* 12(1)**,** 424. doi: 10.1186/s13287-021-02449-9.

Yang, B.C., Kuang, M.J., Kang, J.Y., Zhao, J., Ma, J.X., and Ma, X.L. (2020). Human umbilical cord mesenchymal stem cell-derived exosomes act via the miR-1263/Mob1/Hippo signaling pathway to prevent apoptosis in disuse osteoporosis. *Biochem Biophys Res Commun* 524(4)**,** 883-889. doi: 10.1016/j.bbrc.2020.02.001.

Yang, C., Gao, C., Liu, N., Zhu, Y., Zhu, X., Su, X., et al. (2021). The effect of traumatic brain injury on bone healing from a novel exosome centered perspective in a mice model. *J Orthop Translat* 30**,** 70-81. doi: 10.1016/j.jot.2021.09.003.

Yu, W., Su, X., Li, M., Wan, W., Li, A., Zhou, H., et al. (2021). Three-dimensional mechanical microenvironment enhanced osteogenic activity of mesenchymal stem cells-derived exosomes. *Chemical Engineering Journal* 417. doi: 10.1016/j.cej.2020.128040.

Zavatti, M., Beretti, F., Casciaro, F., Bertucci, E., and Maraldi, T. (2020). Comparison of the therapeutic effect of amniotic fluid stem cells and their exosomes on monoiodoacetate-induced animal model of osteoarthritis. *Biofactors* 46(1)**,** 106-117. doi: 10.1002/biof.1576.

Zha, Y., Li, Y., Lin, T., Chen, J., Zhang, S., and Wang, J. (2021). Progenitor cell-derived exosomes endowed with VEGF plasmids enhance osteogenic induction and vascular remodeling in large segmental bone defects. *Theranostics* 11(1)**,** 397-409. doi: 10.7150/thno.50741.

Zhai, M., Zhu, Y., Yang, M., and Mao, C. (2020). Human Mesenchymal Stem Cell Derived Exosomes Enhance Cell-Free Bone Regeneration by Altering Their miRNAs Profiles. *Adv Sci (Weinh)* 7(19)**,** 2001334. doi: 10.1002/advs.202001334.

Zhang, B., Huang, J., Liu, J., Lin, F., Ding, Z., and Xu, J. (2021a). Injectable composite hydrogel promotes osteogenesis and angiogenesis in spinal fusion by optimizing the bone marrow mesenchymal stem cell microenvironment and exosomes secretion. *Mater Sci Eng C Mater Biol Appl* 123**,** 111782. doi: 10.1016/j.msec.2020.111782.

Zhang, D., Wu, Y., Li, Z., Chen, H., Huang, S., Jian, C., et al. (2021b). MiR-144-5p, an exosomal miRNA from bone marrow-derived macrophage in type 2 diabetes, impairs bone fracture healing via targeting Smad1. *J Nanobiotechnology* 19(1)**,** 226. doi: 10.1186/s12951-021-00964-8.

Zhang, F.X., Liu, P., Ding, W., Meng, Q.B., Su, D.H., Zhang, Q.C., et al. (2021c). Injectable Mussel-Inspired highly adhesive hydrogel with exosomes for endogenous cell recruitment and cartilage defect regeneration. *Biomaterials* 278**,** 121169. doi: 10.1016/j.biomaterials.2021.121169.

Zhang, J., Liu, X., Li, H., Chen, C., Hu, B., Niu, X., et al. (2016). Exosomes/tricalcium phosphate combination scaffolds can enhance bone regeneration by activating the PI3K/Akt signaling pathway. *Stem Cell Res Ther* 7(1)**,** 136. doi: 10.1186/s13287-016-0391-3.

Zhang, J.Y., Rong, Y.L., Luo, C.Y., and Cui, W.D. (2020a). Bone marrow mesenchymal stem cell-derived exosomes prevent osteoarthritis by regulating synovial macrophage polarization. *Aging-US* 12**,** 25138-25152. doi: 10.18632/aging.104110.

Zhang, L., Jiao, G., Ren, S., Zhang, X., Li, C., Wu, W., et al. (2020b). Exosomes from bone marrow mesenchymal stem cells enhance fracture healing through the promotion of osteogenesis and angiogenesis in a rat model of nonunion. *Stem Cell Res Ther* 11(1)**,** 38. doi: 10.1186/s13287-020-1562-9.

Zhang, L., Wang, Q., Su, H., and Cheng, J. (2021d). Exosomes from adipose derived mesenchymal stem cells alleviate diabetic osteoporosis in rats through suppressing NLRP3 inflammasome activation in osteoclasts. *J Biosci Bioeng* 131(6)**,** 671-678. doi: 10.1016/j.jbiosc.2021.02.007.

Zhang, S., Chuah, S.J., Lai, R.C., Hui, J.H.P., Lim, S.K., and Toh, W.S. (2018a). MSC exosomes mediate cartilage repair by enhancing proliferation, attenuating apoptosis and modulating immune reactivity. *Biomaterials* 156**,** 16-27. doi: 10.1016/j.biomaterials.2017.11.028.

Zhang, S., Teo, K.Y.W., Chuah, S.J., Lai, R.C., Lim, S.K., and Toh, W.S. (2019a). MSC exosomes alleviate temporomandibular joint osteoarthritis by attenuating inflammation and restoring matrix homeostasis. *Biomaterials* 200**,** 35-47. doi: 10.1016/j.biomaterials.2019.02.006.

Zhang, T., Jiang, M., Yin, X., Yao, P., and Sun, H. (2021e). Mechanism of Exosomes Involved in Osteoimmunity Promoting Osseointegration Around Titanium Implants With Small-Scale Topography. *Front Bioeng Biotechnol* 9**,** 682384. doi: 10.3389/fbioe.2021.682384.

Zhang, Y., Cai, F., Liu, J., Chang, H., Liu, L., Yang, A., et al. (2018b). Transfer RNA-derived fragments as potential exosome tRNA-derived fragment biomarkers for osteoporosis. *Int J Rheum Dis* 21(9)**,** 1659-1669. doi: 10.1111/1756-185X.13346.

Zhang, Y., Hao, Z., Wang, P., Xia, Y., Wu, J., Xia, D., et al. (2019b). Exosomes from human umbilical cord mesenchymal stem cells enhance fracture healing through HIF-1alpha-mediated promotion of angiogenesis in a rat model of stabilized fracture. *Cell Prolif* 52(2)**,** e12570. doi: 10.1111/cpr.12570.

Zhang, Y., Xie, Y., Hao, Z., Zhou, P., Wang, P., Fang, S., et al. (2021f). Umbilical Mesenchymal Stem Cell-Derived Exosome-Encapsulated Hydrogels Accelerate Bone Repair by Enhancing Angiogenesis. *ACS Appl Mater Interfaces* 13(16)**,** 18472-18487. doi: 10.1021/acsami.0c22671.

Zhao, P., Xiao, L., Peng, J., Qian, Y.Q., and Huang, C.C. (2018). Exosomes derived from bone marrow mesenchymal stem cells improve osteoporosis through promoting osteoblast proliferation via MAPK pathway. *Eur Rev Med Pharmacol Sci* 22**,** 3962-3970. doi: 10.26355/eurrev-201806-15280.

Zhao, Q., Zhang, Y., Xiao, L., Lu, H., Ma, Y., Liu, Q., et al. (2021). Surface engineering of titania nanotubes incorporated with double-layered extracellular vesicles to modulate inflammation and osteogenesis. *Regen Biomater* 8(3)**,** rbab010. doi: 10.1093/rb/rbab010.

Zhi, F., Ding, Y., Wang, R., Yang, Y., Luo, K., and Hua, F. (2021). Exosomal hsa_circ_0006859 is a potential biomarker for postmenopausal osteoporosis and enhances adipogenic versus osteogenic differentiation in human bone marrow mesenchymal stem cells by sponging miR-431-5p. *Stem Cell Res Ther* 12(1)**,** 157. doi: 10.1186/s13287-021-02214-y.

Zhou, J., Liu, H.X., Li, S.H., Gong, Y.S., Zhou, M.W., Zhang, J.H., et al. (2019). Effects of human umbilical cord mesenchymal stem cells-derived exosomes on fracture healing in rats through the Wnt signaling pathway. *Eur Rev Med Pharmacol Sci* 23**,** 4954-4960. doi: 10.26355/eurrev_201906_18086.

Zhu, M., Liu, Y., Qin, H., Tong, S., Sun, Q., Wang, T., et al. (2021). Osteogenically-induced exosomes stimulate osteogenesis of human adipose-derived stem cells. *Cell Tissue Bank* 22(1)**,** 77-91. doi: 10.1007/s10561-020-09867-8.

Zhu, Y., Wang, Y., Zhao, B., Niu, X., Hu, B., Li, Q., et al. (2017). Comparison of exosomes secreted by induced pluripotent stem cell-derived mesenchymal stem cells and synovial membrane-derived mesenchymal stem cells for the treatment of osteoarthritis. *Stem Cell Res Ther* 8(1)**,** 64. doi: 10.1186/s13287-017-0510-9.
